# Genetic association between PCSK9 and coronary artery calcification mediated by inflammatory cytokines

**DOI:** 10.3389/fcvm.2026.1767013

**Published:** 2026-02-16

**Authors:** Weijian Wang, Jiangping Ye, Xinyi Hu, Zongzheng Chen, Li Wang, Liang Chen

**Affiliations:** 1 Department of Cardiology, The 904th Hospital of Joint Logistic Support Force of P.L.A., Wuxi, Jiangsu, China; 2 Department of Emergency, Changzhou Second People’s Hospital of Nanjing Medical University, Changzhou, Jiangsu, China; 3 Department of Cardiology, Wuxi Clinical College of Anhui Medical University, Wuxi, Jiangsu, China

**Keywords:** calcification models, coronary artery calcification, inflammatory cytokines, mediation analysis, Mendelian randomization, PCSK9

## Abstract

**Background:**

Coronary artery calcification (CAC), a hallmark of coronary atherosclerosis, links closely to dysregulated lipid metabolism and chronic inflammation. Proprotein convertase subtilisin/kexin type 9 (PCSK9) inhibitors exert potent lipid-lowering and anti-inflammatory effects, holding translational potential for vascular calcification intervention. However, evidence on PCSK9 inhibition's impact on vascular calcification remains inconsistent. Here, we combined genetic causal analysis with *in vivo*/*in vitro* experiments to explore the therapeutic efficacy of PCSK9 inhibition against CAC and the mediating role of fibroblast growth factor 23 (FGF23) in this process.

**Methods:**

First, we used two-sample Mendelian randomization (MR) and multivariable Mendelian randomization to identify lipid profiles genetically associated with coronary artery calcification. Subsequently, we investigated the value of the PCSK9 gene as a potential therapeutic target for CAC through drug target MR and colocalization analysis, and screened for potential inflammatory mediators via Mediation MR analyses. Following the completion of the aforementioned analyses, we verified the beneficial effect of PCSK9 inhibitors on delaying vascular calcification through animal experiments and cell experiments.

**Results:**

MR analysis revealed that genetic proxies for apolipoprotein B (ApoB) (OR=1.64; 95%CI: 1.42–1.90; *p* < 0.001) and low-density lipoprotein cholesterol (LDL-C) (OR=1.78; 95%CI: 1.50–2.51; *p* < 0.001) were positively causally associated with increased coronary artery calcification (CAC) severity. Drug target MR analysis identified *PCSK9* as a promising CAC therapeutic target (OR=1.19; 95%CI: 1.11–1.27; *p* < 0.001), and colocalization analysis confirmed shared genetic causality between *PCSK9* expression and CAC susceptibility. Mediation MR analysis suggested FGF23 as a partial mediator in the PCSK9-CAC axis (mediated effect=0.024; mediation proportion=13.86%). In animal experiments, calcification upregulated PCSK9 levels (*p* < 0.001), calcification-related proteins (BMP2, BMP4, RUNX2) (*p* < 0.001), and FGF23 expression (*p* < 0.05) versus controls, with all indicators reduced by evolocumab treatment (*p* < 0.001, *p* < 0.05, *p* < 0.05, respectively). In HASMCs, calcification elevated PCSK9 levels (*p* < 0.001), calcification-related proteins (*p* < 0.05), and FGF23 expression (*p* < 0.001), whereas siRNA reversed these changes (*p* < 0.001, *p* < 0.01, *p* < 0.05, respectively).

**Conclusions:**

Inhibition of PCSK9 may effectively slow the progression of coronary artery calcification, with inflammatory mediators such as FGF23 playing key regulatory roles in this process.

## Introduction

Coronary artery calcification (CAC) is a key pathophysiological hallmark of atherosclerosis and serves as a strong predictor of future adverse cardiovascular events (1). Vascular calcification predominantly occurs in the intima and media, with intimal calcification being closely linked to atherosclerotic progression. It represents an active pathological process driven by cardiovascular risk factors and inflammation, involving cell death, matrix vesicle release, cholesterol deposition, and phenotypic transition of smooth muscle cells into chondrocyte-like cells, ultimately leading to abnormal calcium salt deposition in the arterial intima (2). In addition to low-density lipoprotein cholesterol (LDL-C), other atherogenic lipid components—such as apolipoprotein B (ApoB), non-high-density lipoprotein cholesterol (non-HDL-C), remnant cholesterol, and lipoprotein(a) [Lp(a)] —have also been shown to be independently associated with the occurrence and progression of CAC. These findings underscore the importance of comprehensive lipid profiling in identifying individuals at high risk of calcification (3, 4).

Proprotein convertase subtilisin/kexin type 9 (PCSK9) is a protease primarily secreted by the liver that promotes degradation of low-density lipoprotein receptors (LDLR), thereby increasing circulating LDL-C levels and contributing to the pathogenesis of atherosclerotic cardiovascular disease (ASCVD). Beyond its lipid-regulating role, PCSK9 is also linked to inflammation, as its inhibition reduces the expression of pro-inflammatory mediators such as tumor necrosis factor-α (TNF-α), interleukin-1β (IL-1β), and nucleotide-binding oligomerization domain-like receptor family pyrin domain-containing 3 (NLRP3) (5). However, current evidence regarding the effect of PCSK9 inhibition on vascular calcification remains inconclusive (6–8). Using Mendelian randomization (MR), the present study investigates the causal relationship between common lipid traits and CAC, evaluates the potential therapeutic implications of PCSK9 inhibition, and further explores the mediating role of inflammatory cytokines in this process.

## Materials and methods

### Study design

MR leverages the random allocation of genetic variants at conception as instrumental variables (IVs) to estimate causal relationships between exposures and outcomes. Because these variants are randomly assigned and largely independent of environmental influences, MR analyses are relatively robust against unmeasured confounding and reverse causality (9, 10).

This study strictly adhered to the three core assumptions of MR. First, the relevance assumption requires that selected genetic variants are strongly associated with the exposure, thereby serving as valid proxies. Second, the independence assumption states that, conditional on the exposure, the genetic variants remain independent of confounders affecting the outcome. Third, the exclusion restriction assumption requires that genetic variants influence the outcome only through the exposure, without direct or exposure-independent indirect pathways.

**Figure 1:**
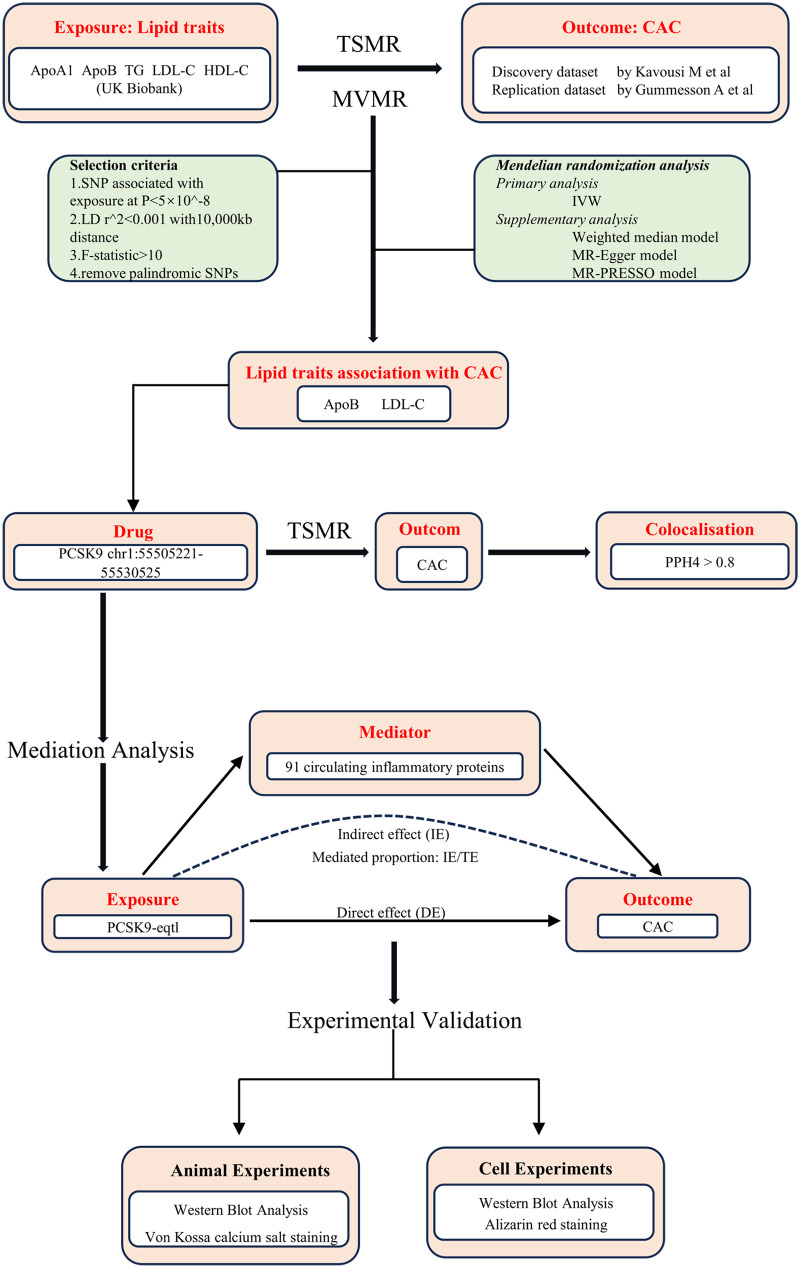
Overview of the study design. ApoA1, apolipoprotein A-I; ApoB, apolipoprotein B; HDL-C, high density lipoprotein cholesterol; LDL-C, low density lipoprotein cholesterol; TG, triglyceride; CAC, coronary artery calcification; TSMR, two-sample Mendelian randomization; MVMR, multivariable Mendelian randomization; SNPs, single nucleotide polymorphisms; LD, linkage disequilibrium; IVW, inverse variance weighted; PCSK9, proprotein convertase subtilisin/kexin type 9; eQTL, expression quantitative trait loci; PPH4, Posterior Probability of Hypothesis 4; IE, indirect effect; DE, direct effect.

Based on these principles, we employed a two-sample MR design to examine the potential causal relationships between five common lipid traits, inflammatory cytokines, and CAC. To verify statistical power, analysis of the Mendelian randomization was performed using the dedicated online tool mRnd (https://shiny.cnsgenomics.com/mRnd/). Pleiotropy and heterogeneity tests were performed to ensure the robustness of the findings, while Steiger tests and reverse MR analyses were conducted to exclude reverse causality. To account for correlations among lipid traits, we applied multivariable MR to identify independent exposures associated with CAC. Furthermore, drug-target MR and colocalization analyses were used to evaluate the therapeutic relevance of the PCSK9 gene in CAC. Mediation MR analyses were then performed to identify potential inflammatory mediators linking PCSK9 to CAC. Finally, animal and cellular experiments were conducted to validate the effects of PCSK9 inhibition on vascular calcification. An overview of the study design is presented in Figure 1.

All animal procedures were carried out in accordance with the Guide for the Care and Use of Laboratory Animals of the National Institutes of Health (NIH) and were approved by the Animal Ethics Committee of the 904th Hospital of the Joint Logistic Support Force of the Chinese People's Liberation Army.

### Data sources

Genome-wide association study (GWAS) data for lipid traits were obtained from the IEU OpenGWAS database (https://gwas.mrcieu.ac.uk) and included apolipoprotein A1 (ApoA1), ApoB, high-density lipoprotein cholesterol (HDL-C), LDL-C, and triglycerides (TG), with corresponding GWAS IDs ieu-b-107 to ieu-b-111 (11). Data on circulating inflammation-related proteins were derived from a meta-analysis of 91 inflammatory proteins across 11 cohorts, comprising 14,824 participants of European ancestry (12). Full GWAS summary statistics for these proteins are available in the GWAS Catalog (https://www.ebi.ac.uk/gwas/) under accession numbers GCST90274758 to GCST90274848. Summary statistics for CAC were obtained from a multi-ancestry GWAS meta-analysis (13), but only data from participants of European ancestry were used to minimize population stratification bias (GWAS Catalog accession number: GCST90278456). An additional CAC dataset (GCST90503074) was employed for replication analyses (14). For mediation MR analyses, PCSK9 expression quantitative trait locus (eQTL) data were used as the exposure, extracted from the GTEx V8 database (https://gtexportal.org/) via the R package xQTLbiolinks (15). A complete summary of all GWAS datasets used in this study is provided in Table 1.

**Table 1:** Summary of genome-wide association studies (GWAS) datasets in our study.

Phenotype	GWAS ID	Author, published year	Consortium	Sample size	Population
Exposure					
ApoA1	ieu-b-107	Richardson TG et al, 2020	UK Biobank	393,193	European
ApoB	ieu-b-108	Richardson TG et al, 2020	UK Biobank	439,214	European
HDL-C	ieu-b-109	Richardson TG et al, 2020	UK Biobank	403,943	European
LDL-C	ieu-b-110	Richardson TG et al, 2020	UK Biobank	440,546	European
TG	ieu-b-111	Richardson TG et al, 2020	UK Biobank	441,016	European
Outcome					
CAC	GCST90278456	Kavousi M et al, 2023	NA	28,655	European
CAC	GCST90503074	Gummesson A et al, 2025	NA	26,000	European
Mediation					
91 inflammatory factors	GCST90274758 - GCST90274848	Zhao JH et al, 2023	NA	14,736	European

ApoA1, apolipoprotein A-I; ApoB, apolipoprotein B; HDL-C, high density lipoprotein cholesterol; LDL-C, low density lipoprotein cholesterol; TG, triglyceride; CAC, coronary artery calcification.

### Genetic variant selection

In the MR analysis, single nucleotide polymorphisms (SNPs) strongly associated with the exposures were selected as IVs. For the five lipid traits and 91 inflammatory traits, SNPs were identified using a genome-wide significance threshold of *p* < 5 × 10^−8^. To ensure independence, SNPs in linkage disequilibrium (LD) were excluded using a clumping threshold of 10,000 kb and *r^2^* < 0.001.The strength of each IV was assessed using the F-statistic, calculated as: *F* = *R*^2^ (*N* – 2)/(1 – *R*^2^), where *R*^2^ = 2*β*^2^ × EAF × (1 – EAF), with *β* representing the SNP effect size on the exposure and EAF the effect allele frequency. An F-statistic > 10 was considered indicative of sufficient instrument strength. SNPs failing to meet this criterion were excluded, and no proxy SNPs were used. The Steiger test was performed to confirm the causal direction, thereby minimizing bias from reverse causality. For drug-target MR analyses, SNPs located within 1 Mb upstream and downstream of the PCSK9 locus were extracted from GWAS datasets of lipid traits. Here, a clumping threshold of 100 kb and *r^2^* < 0.3 was applied. In addition, PCSK9-eQTL data were obtained from the GTEx V8 database, restricted to the “Whole Blood” tissue type, with a stringent threshold of *p* < 5 × 10^−8^ for SNP selection.

### MR analysis and mediation analysis

We conducted MR analyses using multiple approaches, with inverse variance weighting (IVW) as the primary method. The IVW approach provides robust causal estimates under the assumption that all genetic variants are valid instrumental variables and is capable of accommodating heterogeneity among SNPs. Heterogeneity was assessed using Cochran's *Q* test, where a *p*-value < 0.05 indicates significant heterogeneity and suggests that a fixed-effects model may be inappropriate. Outlier detection was performed using the MR-pleiotropy residual sum and outlier (MR-PRESSO) test, and, after outlier removal, corrected causal estimates were obtained via fixed-effects IVW. Additionally, the MR-Egger intercept was used to evaluate horizontal pleiotropy across the instrumental variables, acknowledging that this method has reduced statistical power.

To explore potential reverse causality, we performed two-sample MR treating CAC as the exposure and lipid traits as the outcomes. Lipid traits demonstrating a causal effect on CAC in these analyses were subsequently incorporated into multivariable Mendelian Randomization (MVMR). In MVMR, IVW was primarily employed, and exposures with *p*-value < 0.05 were considered independent factors associated with the outcome.

In the Drug Target MR analysis, variants from GWAS datasets of relevant lipid traits were selected as IVs. These variants were located within a 1 Mb window upstream and downstream of the PCSK9 gene (chromosomal location: chr1:55,505,221–55,530,525). The selected IVs were used to assess the association between lipid traits and CAC. Causal effect estimates were obtained using the IVW method, with a *p*-value < 0.05 considered statistically significant. Additionally, colocalization analysis was performed to determine whether the genetic association signals for the exposure and outcome traits originated from the same causal variant. This step enhances the credibility of causal inference by confirming that observed associations are driven by a shared causal variant rather than distinct variants within overlapping genomic regions.

Furthermore, we conducted a two-step Mendelian Randomization mediation analysis to explore potential pathways linking PCSK9 expression to CAC. PCSK9-eQTL data were treated as the “exposure,” inflammatory cytokines as the “mediator,” and CAC as the “outcome.” First, TSMR was performed to estimate the total effect of the exposure on the outcome. Subsequently, stepwise analyses were conducted to evaluate the effect of the exposure on the mediator and the effect of the mediator on the outcome, respectively. A mediating effect was considered present only if IVW analyses in both steps yielded *p*-values < 0.05. The total effect of PCSK9-eQTL on CAC was decomposed into direct and indirect effects, and the proportion of the total effect mediated by the inflammatory cytokines was calculated as the ratio of the indirect effect to the total effect. The 95% confidence interval for the mediating effect was estimated using the delta method.

### Establishment and grouping of animal models

Twenty male 8-week-old specific pathogen-free (SPF) C57BL/6 mice, weighing 20–25 g, were obtained from Qinglongshan Animal Breeding Farm (Jiangning District, Nanjing, China). Mice were housed in individual cages with *ad libitum* access to food and water under controlled conditions (22–26°C, 40%–60% relative humidity, 12 h light/12 h dark cycle).

After one week of acclimatization, mice were randomly assigned to four groups (*n* = 5 per group): (1) Control group, (2) Evolocumab group, (3) Calcification group, and (4) Calcification + Evolocumab group. Mice in the Evolocumab and Calcification + Evolocumab groups received subcutaneous injections of Evolocumab (GLPEIO, USA) at 10 mg/kg every two weeks.

Calcification was induced in the Calcification and Calcification + Evolocumab groups using a combined regimen. Vitamin D_3_ in absolute ethanol (3 × 10^5^ U/mL) was intramuscularly injected into the right femoral region at 0.001 mL/g body weight, followed by local massage to enhance absorption. Simultaneously, nicotine in peanut oil (10 mg/mL) was administered via oral gavage at 0.0025 mL/g body weight, with a second dose given 9 h later. Thereafter, mice in the calcification groups were maintained on a long-term high-fat diet.

At the end of the 8-week experimental period, mice were anesthetized with an intraperitoneal injection of 3% sodium pentobarbital (45 mg/kg). After hair removal, the thoracic and abdominal cavities were opened, and the heart along with the ascending aorta (from the aortic root to the bifurcation of the abdominal aorta) were carefully isolated for subsequent analyses.

### Cell culture and treatment

Human aortic smooth muscle cells (HASMCs) were obtained from Shanghai Fuheng Biotechnology Co., Ltd. (Shanghai, China) and cultured in complete medium for HASMCs (Fuheng Biology, Shanghai, China) supplemented with 10% heat-inactivated fetal bovine serum, penicillin, streptomycin, and growth factors. Cells were maintained in a humidified incubator at 37°C with 5% CO₂.

Human PCSK9 small interfering RNA (siRNA) and negative control siRNA (si-NC) were designed and synthesized by Heyuan Biotechnology Co., Ltd. (Shanghai, China). Experiments were organized into four groups: (1) Control group, (2) si-NC group, (3) Calcification group, and (4) Calcification + PCSK9 siRNA knockdown (Calcification + siPCSK9) group. HASMCs at passages 3–5 were evenly seeded into 12-well plates. When cell confluency reached 40∼50%, siRNA and Lipofectamine® 3000 reagent (Life Technologies) were separately diluted in serum-free, antibiotic-free Opti-MEM medium. The two solutions were then combined, incubated at room temperature for 20 min, and added to the wells to transfect cells in the si-NC and Calcification + siPCSK9 groups. Transfection was allowed to proceed for 48 h in a 37°C, 5% CO₂ incubator.

Following transfection, cells in the Calcification and Calcification + siPCSK9 groups were treated with 10 mmol/L β-glycerophosphate and 7.2 mmol/L calcium chloride to induce calcification for 14 days, with medium replacement every 2 days, thereby completing the cell model experiment.

### Von Kossa calcium salt staining

The aorta was carefully excised from C57BL/6 mice, and connective tissue, surrounding fat, and residual blood within the lumen were removed using phosphate-buffered saline (PBS). After blotting with filter paper, the aortic arch was collected and processed for cross-sectional analysis. Tissue samples were routinely dehydrated, embedded in paraffin, and sectioned into 4 μm slices using a paraffin microtome (Leica UC7, Leica). Sections were mounted on slides and dried. Deparaffinization and rehydration were performed sequentially using xylene and graded ethanol solutions. Calcification was then assessed by Von Kossa staining according to the manufacturer's protocol (Servicebio, Wuhan, China), and aortic calcification was examined under a light microscope.

### Alizarin red staining

After 14 days of calcification induction, HASMCs were rinsed three times with ice-cold PBS and fixed with paraformaldehyde for 15 min, followed by three additional PBS washes. Cells were then stained with 1% alizarin red S solution (pH 4.2; Solarbio, Beijing, China) at room temperature for 20 min. After three washes with PBS, calcified nodules were visualized and imaged under a light microscope.

### Western blot analysis

Mouse aorta and HASMC samples were lysed with RIPA buffer containing protease and phosphatase inhibitors. Protein supernatants were collected by thorough agitation and centrifugation at low temperature, and protein concentrations were determined using a BCA assay kit (Beyotime Biotechnology, Shanghai, China). Equal amounts of protein were separated by SDS–polyacrylamide gel electrophoresis and transferred onto polyvinylidene fluoride (PVDF) membranes. Membranes were blocked with 5% skim milk for 2 h at room temperature and incubated overnight at 4°C with the corresponding primary antibodies: PCSK9 (Immunoway, YT7913, 1:1000), BMP2 (Immunoway, YT0498, 1:1000), BMP4 (Immunoway, YT7841, 1:1000), RUNX2 (Immunoway, YM8347, 1:1000), FGF23 (Immunoway, YN6045, 1:1000). Membranes were then incubated for 2 h at room temperature with goat anti-rabbit IgG (H + L)-FITC (Abways, AB0101, 1:5000). Protein signals were detected using an enhanced chemiluminescence system (ECL; Biosharp, Hefei, China) and imaged with an exposure device (Analytikjena, Shanghai, China). Band intensities were quantified using ImageJ software, and protein expression levels were normalized to GAPDH (Abways, AB0037, 1:15,000).

### Statistical analysis

Mendelian randomization analyses were performed in R 4.3.3 using the packages *TwoSampleMR*, *MRPRESSO*, and *coloc*. Results are presented as effect sizes (ES) with corresponding 95% confidence intervals (CI). Visualization was conducted using the R packages *locuscomparer* and *forestploter*. Other statistical analyses were performed using SPSS 26.0 and GraphPad Prism 9. Normality of data was assessed using the Shapiro–Wilk test. Continuous variables with normal distribution are expressed as mean ± standard deviation and compared using Student's *t*-test, while non-normally distributed variables were analyzed with the Mann–Whitney *U* test. Differences among multiple groups were evaluated by one-way analysis of variance (ANOVA). All tests were two-tailed, and *p*-values < 0.05 were considered statistically significant.

## Results

### Exploration of the causal effect of lipid traits on coronary artery calcification

We employed the IVW method as the primary approach for MR analysis and conducted a TSMR study to investigate the causal relationship between common lipid traits and CAC. At a significance threshold of *p* < 0.05, ApoB, HDL-C, LDL-C, and TG were found to be causally associated with CAC. Specifically, ApoB, LDL-C, and TG were linked to an increased risk of CAC, whereas HDL-C was associated with a decreased risk; detailed results are presented in Table 2. Heterogeneity and pleiotropy analyses indicated that all these lipid traits passed the corresponding tests (Table 3).

**Table 2:** Mendelian randomization results of lipid traits with risk of coronary artery calcification (univariable).

Lipid trait	Methods	CAC (discovery dataset)		CAC (replication dataset)	
		OR (95%CI)	*P* value	OR (95%CI)	*P* value
ApoA1	MR Egger	1.09 (0.82, 1.44)	0.56	0.96 (0.88, 1.04)	0.34
	Weighted median	0.93 (0.76, 1.15)	0.52	0.95 (0.89, 1.01)	0.10
	IVW	0.95 (0.83, 1.08)	0.43	0.98 (0.93, 1.02)	0.28
ApoB	MR Egger	1.94 (1.47, 2.56)	<0.001	1.29 (1.19, 1.39)	<0.001
	Weighted median	1.65 (1.35, 2.03)	<0.001	1.27 (1.18, 1.36)	<0.001
	IVW	1.64 (1.42, 1.90)	<0.001	1.25 (1.19, 1.31)	<0.001
HDL-C	MR Egger	0.93 (0.69, 1.25)	0.64	0.96 (0.89, 1.04)	0.36
	Weighted median	0.79 (0.65, 0.96)	0.02	0.94 (0.89, 1.01)	0.07
	IVW	0.78 (0.68, 0.89)	<0.001	0.95 (0.91, 0.99)	0.01
LDL-C	MR Egger	2.12 (1.58, 2.85)	<0.001	1.29 (1.19, 1.40)	<0.001
	Weighted median	1.89 (1.49, 2.40)	<0.001	1.24 (1.15, 1.35)	<0.001
	IVW	1.78 (1.50, 2.51)	<0.001	1.25 (1.18, 1.31)	<0.001
TG	MR Egger	1.20 (0.94, 1.53)	0.15	1.18 (1.09, 1.28)	<0.001
	Weighted median	1.35 (1.10, 1.65)	0.003	1.19 (1.12, 1.27)	<0.001
	IVW	1.35 (1.19, 1.54)	<0.001	1.14 (1.09, 1.19)	<0.001

ApoA1, apolipoprotein A-I; ApoB, apolipoprotein B; HDL-C, high density lipoprotein cholesterol; LDL-C, low density lipoprotein cholesterol; TG, triglyceride; CAC, coronary artery calcification; OR, odds ratio; CI, confidence interval; IVW, Inverse Variance Weighted.

**Table 3:** Heterogeneity and horizontal pleiotropy in the association between lipid traits and coronary artery calcification risk.

Lipid trait		CAC (discovery dataset)						CAC (replication dataset)					
		Heterogeneity			Horizontal pleiotropy			Heterogeneity			Horizontal pleiotropy		
		Cochran'sQ	Q_df	Q_pval	Egger intercept	SE	*P* value	Cochran's Q	Q_df	Q_pval	Egger intercept	SE	*P* value
ApoA1	MR Egger	59.78	56	0.34	−8.00E-03	7.32E-03	0.28	94.54	66	0.01	1.07E-03	2.24E-03	–
	IVW	61.05	57	0.33	–	–	–	94.87	67	0.01	–	–	0.63
ApoB	MR Egger	26.46	36	0.88	−1.10E-02	8.05E-03	0.18	41.02	36	0.26	−2.22E-03	2.29E-03	–
	IVW	28.34	37	0.85	–	–	–	42.10	37	0.26	–	–	0.34
HDL-C	MR Egger	73.41	63	0.17	−9.60E-03	7.41E-03	0.20	64.16	65	0.51	−8.51E-04	1.89E-03	–
	IVW	75.37	64	0.16	–	–	–	64.37	65	0.53	–	–	0.65
LDL-C	MR Egger	22.12	30	0.85	1.19E−02	8.27E-03	0.16	16.84	26	0.91	−2.97E-03	2.67E-03	–
	IVW	24.21	31	0.80				18.08	27	0.90	–	–	0.28
TG	MR Egger	33.74	45	0.89	7.54E−03	6.53E-03	0.25	36.64	38	0.53	−2.58E-03	2.37E-03	-
	IVW	35.08	46	0.88	–	–	–	37.83	39	0.52	–	–	0.28

ApoA1, apolipoprotein A-I; ApoB, apolipoprotein B; HDL-C, high density lipoprotein cholesterol; LDL-C, low density lipoprotein cholesterol; TG, triglyceride; CAC, coronary artery calcification; IVW, Inverse Variance Weighted.

To further account for potential confounding, we performed MVMR analysis. When ApoB, HDL-C, LDL-C, and TG were included simultaneously, no significant causal relationship with CAC was observed (IVW, *p* > 0.05). Notably, the *F*-statistic for each exposure was below 10, suggesting insufficient statistical power. Considering the strong clinical correlation between ApoB and LDL-C, potential collinearity may have affected the analysis when both were included. Therefore, we performed MVMR analyses including only one of them at a time. These analyses confirmed that ApoB and LDL-C were independently associated with CAC, with substantially increased *F*-statistics for each exposure (Table 4).

**Table 4:** Mendelian randomization results of lipid traits with risk of coronary artery calcification (multivariable).

Lipid trait	Methods	CAC (discovery dataset)			CAC (replication dataset)		
		OR (95%CI)	*P* value	F value	OR (95%CI)	*P* value	F value
Variables in MVMR							
ApoB	IVW	1.01 (0.47, 2.11)	0.97	3.92	1.05 (0.83, 1.32)	0.69	4.01
HDL-C	IVW	0.86 (0.70, 1.04)	0.12	8.30	0.97 (0.91, 1.03)	0.30	8.75
LDL-C	IVW	1.65 (0.73, 3.74)	0.23	3.79	1.23 (0.95, 1.59)	0.11	3.88
TG	IVW	1.13 (0.94, 1.35)	0.21	53.00	1.09 (1.03, 1.16)	0.01	60.32
Variables in MVMR							
HDL-C	IVW	0.83 (0.71, 0.97)	0.02	149.28	0.97 (0.92, 1.02)	0.19	163.01
LDL-C	IVW	1.73 (1.46, 2.06)	<0.001	159.80	1.28 (1.21, 1.34)	<0.001	161.71
TG	IVW	1.11 (0.93, 1.32)	0.27	130.72	1.10 (1.04, 1.16)	0.002	139.93
Variables in MVMR							
ApoB	IVW	1.57 (1.33, 1.84)	<0.001	179.59	1.25 (1.19, 1.31)	<0.001	180.69
HDL-C	IVW	0.91 (0.78, 1.06)	0.21	149.32	0.99 (0.94, 1.04)	0.77	162.06
TG	IVW	1.13 (0.94, 1.35)	0.20	121.89	1.09 (1.03, 1.16)	0.004	130.11

ApoB, apolipoprotein B; HDL-C, high density lipoprotein cholesterol; LDL-C, low density lipoprotein cholesterol; TG, triglyceride; CAC, coronary artery calcification; OR, odds ratio; CI, confidence interval; MVMR, multivariable Mendelian randomization; IVW, Inverse Variance Weighted.

To clarify the direction of causality and exclude the possibility of reverse causation, Steiger tests and reverse MR analyses were performed. The results indicated no evidence of reverse causality between ApoB, LDL-C, and CAC. Furthermore, to assess the robustness of our findings, we repeated the two-sample MR and MVMR analyses using an independent CAC dataset (GCST90503074), yielding results consistent with the primary analyses.

### Exploration of the potential role of PCSK9 inhibition in improving coronary artery calcification

PCSK9 inhibitors are primarily used clinically to lower LDL-C but also exert effects on reducing ApoB levels. Based on our previous MR analyses identifying causal relationships between ApoB, LDL-C, and CAC, we further explored the potential therapeutic value of PCSK9 inhibition for CAC.

First, genetic variants located within a 1 Mb window upstream and downstream of the PCSK9 gene were extracted from the LDL-C GWAS dataset, and drug-target MR analyses were performed with CAC as the outcome. The results demonstrated a significant causal association between LDL-C variants targeting PCSK9 and CAC, which was further validated using PCSK9-eQTL data as the exposure.

Colocalization analysis indicated a high degree of overlap between the genetic signals for LDL-C and CAC in the PCSK9 region (PPH4 > 0.95), with the primary driving variant identified as the well-characterized loss-of-function mutation rs11591147. This suggests that the association between LDL-C and CAC may be mediated by the same causal variant, supporting PCSK9 as a potential therapeutic target for modulating CAC.

**Figure 2:**
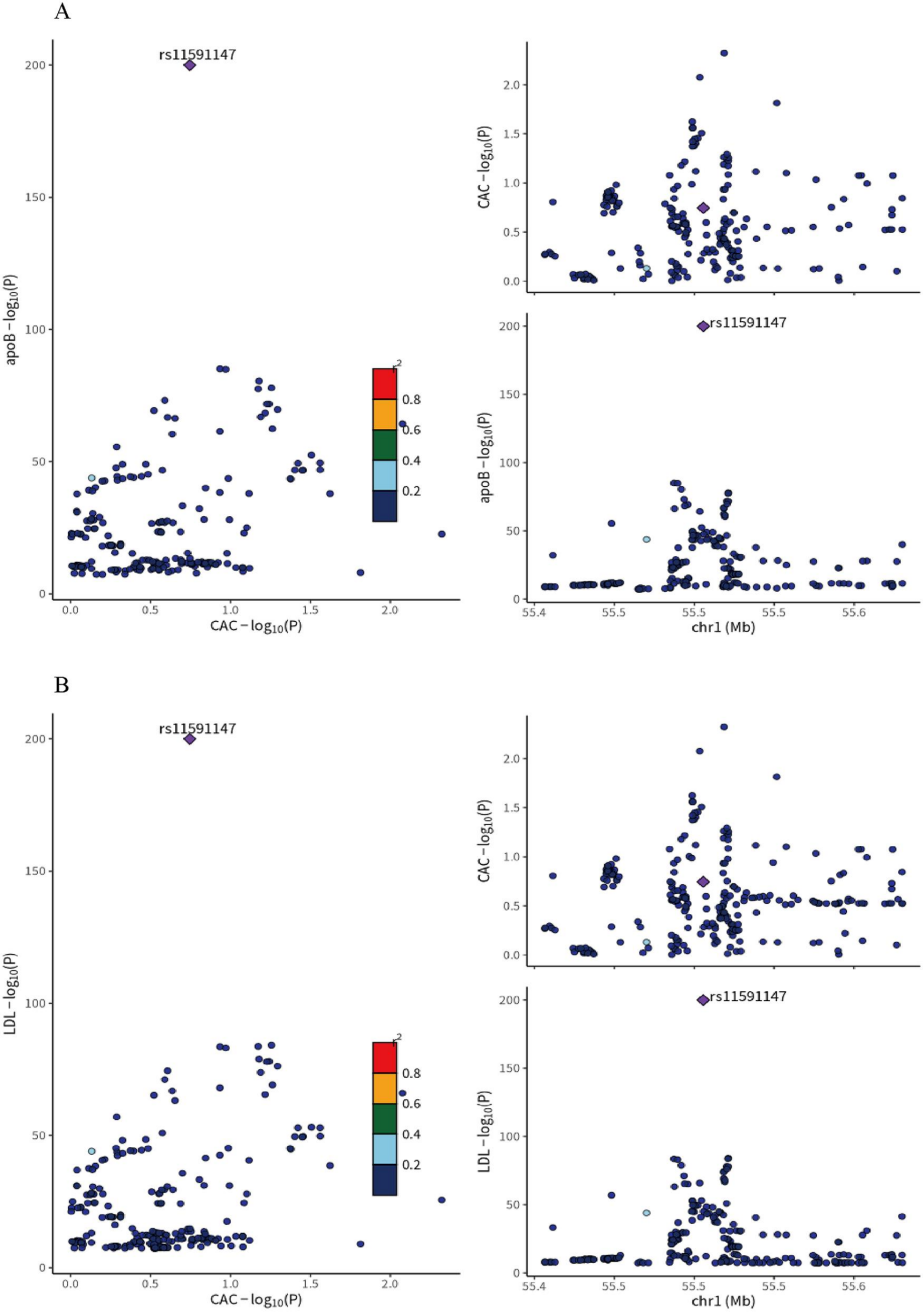
**(A)** colocalization analysis of LDL-C and coronary artery calcification (CAC) GWAS at the PCSK9 locus ±1Mb in whole blood. **(B)** Colocalization analysis of ApoB and Coronary artery calcification (CAC) GWAS at the PCSK9 locus ±1Mb in whole blood. rs11591147(purple dot) represents the top SNP with the minimal sum of *p* value. r2 represents the degree of linkage between SNPs and top SNP. ApoB, apolipoprotein B; LDL-C, low density lipoprotein cholesterol; CAC, coronary artery calcification.

Finally, analyses using the ApoB GWAS dataset yielded similar results, further corroborating these findings (Figure 2).

**Figure 3:**
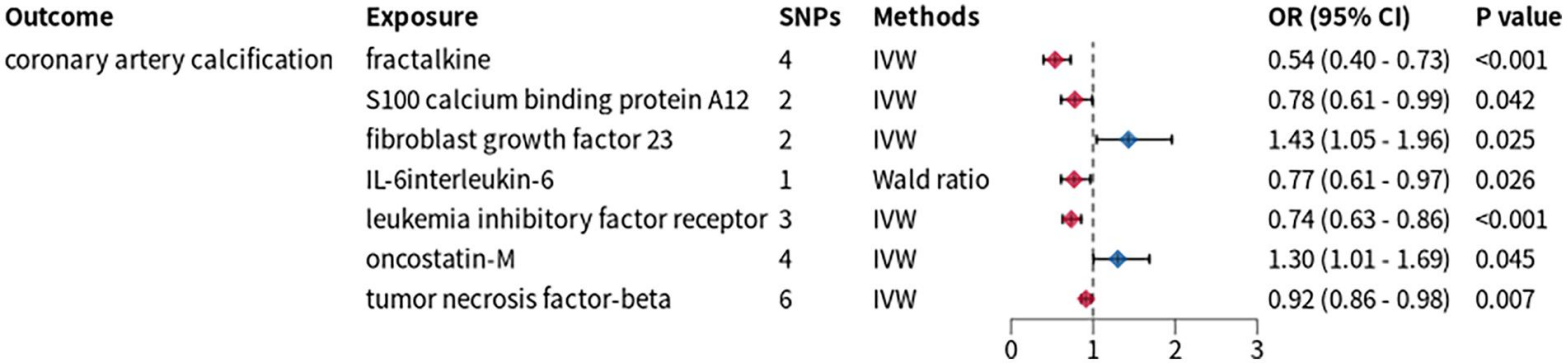
Mendelian randomization analysis between inflammatory cytokines and CAC. SNPs, single nucleotide polymorphisms; OR, odds ratio; CI, confidence interval; IVW, inverse variance weighted.

### Exploration of the relationship between PCSK9 and CAC mediated by inflammatory cytokines

We applied a stringent threshold of *p* < 5 × 10^−8^ to extract IVs from GWAS data of 91 inflammatory proteins, yielding IVs for 74 proteins for TSMR analysis. This analysis revealed causal associations between CAC and the levels of fibroblast growth factor 23 (FGF23), oncostatin-M (OSM), fractalkine (FKN), protein S100-A12, interleukin-6 (IL-6), as well as measurements of leukemia inhibitory factor receptor (LIFR) and tumor necrosis factor-beta (TNF-β) (Figure 3). Subsequently, we conducted an additional two-sample Mendelian randomization analysis with PCSK9-eQTL as the exposure and these seven inflammatory proteins as the outcomes. The results demonstrated that FGF23 and IL-6 exhibited significant causal associations with PCSK9-eQTL.

**Figure 4:**
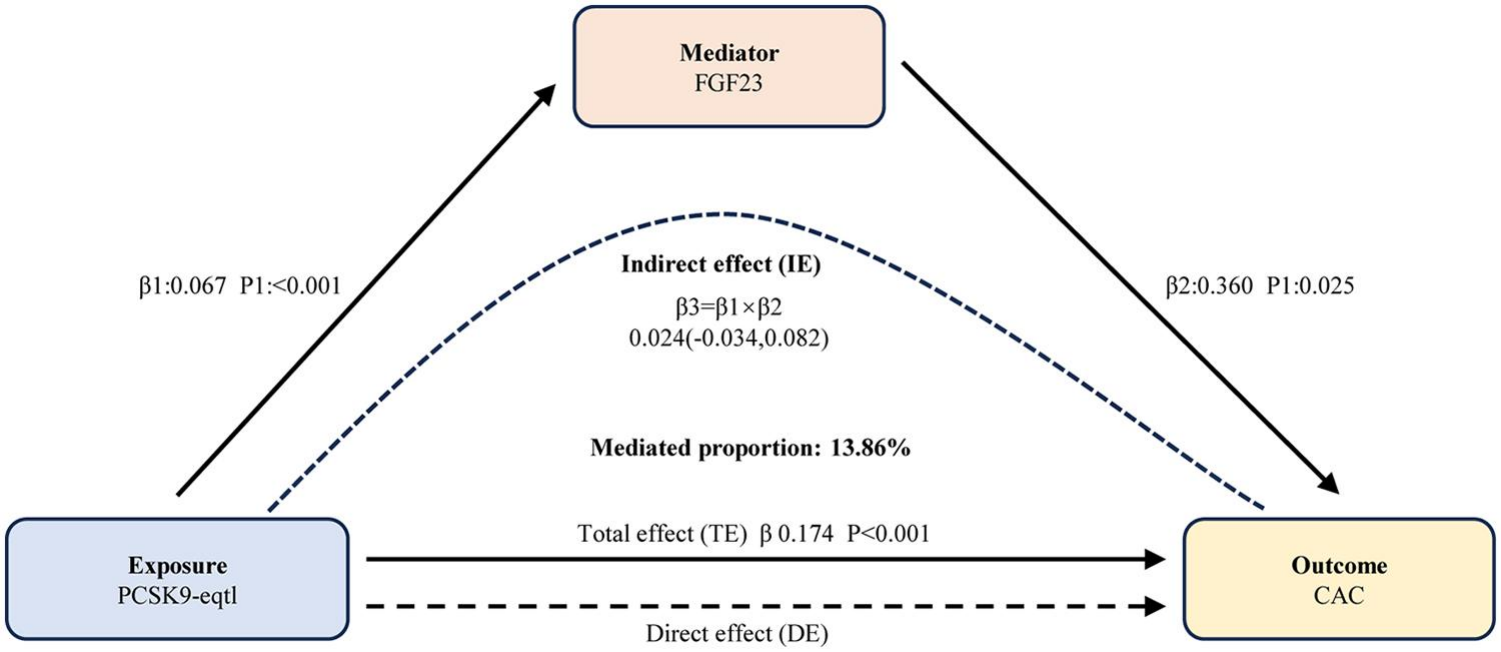
Results of mediation analyses. Elevated PCSK9 levels augment coronary artery calcification quantity partially via FGF23. FGF23, fibroblast growth factor 23; PCSK9, proprotein convertase subtilisin/kexin type 9; eQTL, expression quantitative trait loci; CAC, coronary artery calcification; IE, indirect effect; DE, direct effect; TE, total effect.

We then quantified the mediating effects and their proportions using the delta method (Figure 4). Specifically, the mediating effect of FGF23 accounted for 0.02412, corresponding to 13.86% of the total effect. In contrast, IL-6 exhibited a negative mediating effect proportion with a non-significant *p*-value (*p* > 0.05), indicating no significant mediation. Consequently, IL-6 was not included in subsequent experimental validation.

### Inhibition of PCSK9 reduces the calcification degree of vascular smooth muscle cells

**Figure 5:**
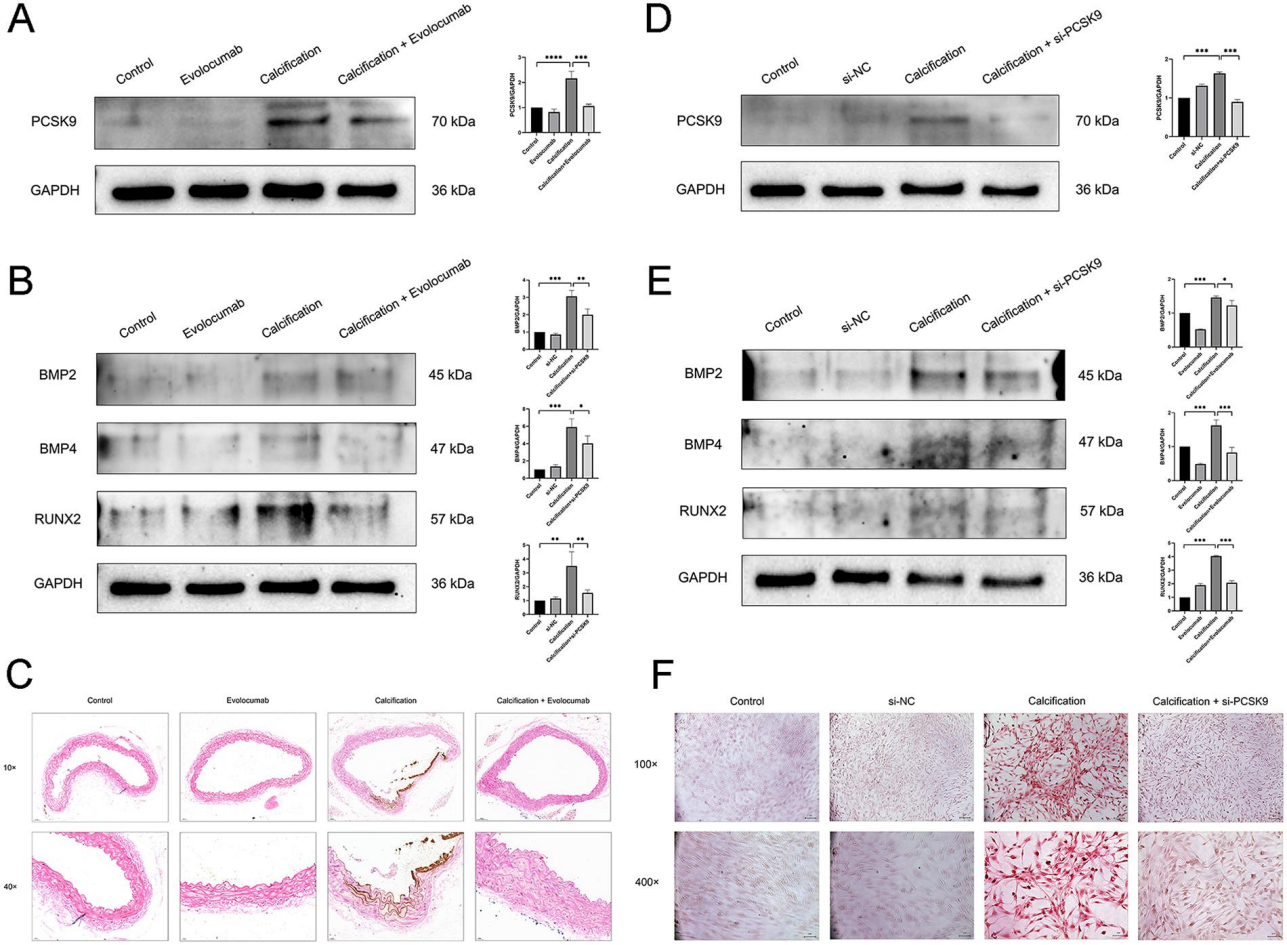
**(A)** Representative western blots of PCSK9 in mouse aortic tissue and their corresponding quantification (*n* = 3 per group). **(B)** Representative Western blots of calcification-related markers (BMP2, BMP4, RUNX2) in mouse aortic tissue and their corresponding quantification (*n* = 3 per group). **(C)** Von Kossa staining images of mouse aortic tissue. 10×: scale bar, 50 μm; 40×: scale bar, 20 μm. **(D)** Representative Western blots of PCSK9 in human aortic smooth muscle cells (HASMCs) and their corresponding quantification (*n* = 3 per group). **(E)** Representative Western blots of calcification-related markers (BMP2, BMP4, RUNX2) in HASMCs and their corresponding quantification (*n* = 3 per group). **(F)** Alizarin Red staining images of HASMCs in each group. 100×: scale bar, 1 px. 400×: scale bar, 1 μm. Data are presented as mean ± standard deviation (SD) from at least three independent experiments. *p < 0.05, **p < 0.01, ***p < 0.001, ****p < 0.0001. PCSK9, proprotein convertase subtilisin/kexin type 9; GAPDH, Glyceraldehyde-3-Phosphate Dehydrogenase; BMP2, Bone Morphogenetic Protein 2; BMP4, Bone Morphogenetic Protein 4; RUNX2, Runt-related Transcription Factor 2.

In animal experiments, we used Evolocumab to inhibit the expression of PCSK9 in mouse blood vessels. As shown in Figure 5A, compared with the control group, the PCSK9 level in mice of the calcification group was significantly increased (*p* < 0.001); while in the calcification + Evolocumab group, the PCSK9 level was significantly decreased compared with the calcification group (*p* < 0.001), suggesting that Evolocumab can effectively inhibit the expression of PCSK9 in mice. Furthermore, detection via Western blot revealed that the expression of calcification-related proteins (BMP2, BMP4, RUNX2) in mice of the calcification group was significantly higher than that in the control group (*p* < 0.001); whereas the above indicators in the calcification + Evolocumab group were significantly lower than those in the calcification group (*p* < 0.05) (Figure 5B). The results of Von Kossa staining also indicated that obvious calcium salt deposition was observed in the aorta of mice in the calcification group, while the degree of deposition was significantly reduced after Evolocumab intervention (Figure 5C).

In cellular experiments, we used siRNA to knock down the expression of PCSK9 in HASMCs. As shown in the Figure 5D, compared with the control group, the PCSK9 level in HASMCs of the calcification group was significantly increased (*p* < 0.001), while the PCSK9 level in the calcification + si-PCSK9 group was significantly decreased compared with the calcification group (*p* < 0.001), indicating that the siRNA intervention was successful. Further Western blot detection showed that the expression of calcification-related proteins in the calcification group was significantly increased (*p* < 0.05), while that in the calcification + si-PCSK9 group was significantly decreased (*p* < 0.01) (Figure 5E). The results of alizarin red staining also confirmed that si-PCSK9 treatment could significantly reduce the calcification degree of HASMCs. In conclusion, inhibiting PCSK9 can effectively alleviate the calcification of vascular smooth muscle cells (Figure 5F).

### FGF23 is involved in the attenuation of calcification induced by PCSK9 inhibition

**Figure 6:**
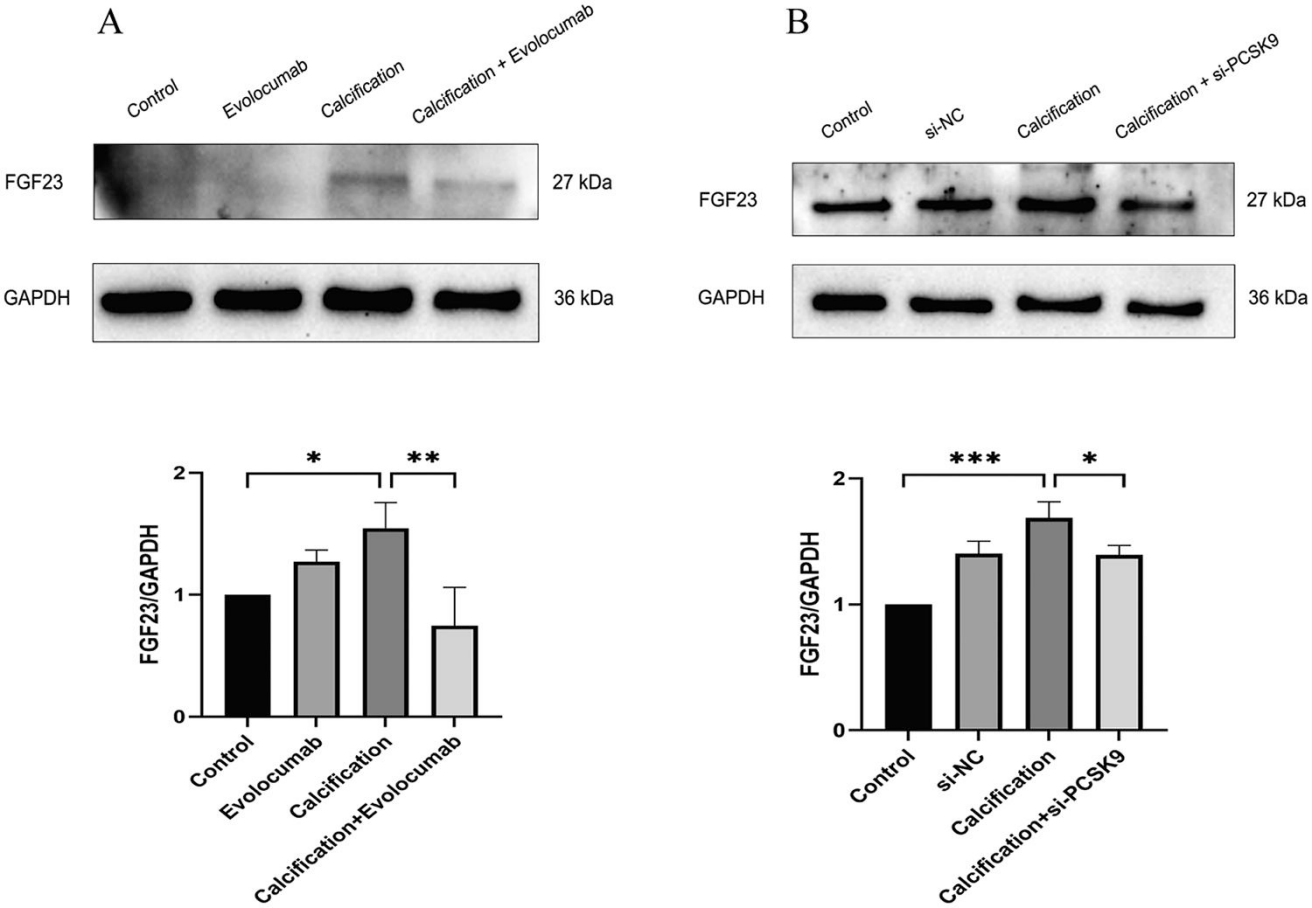
**(A)** representative western blot of FGF23 in mouse aortic tissue and its corresponding quantification (*n* = 3 per group). **(B)** Representative Western blot of FGF23 in human aortic smooth muscle cells (HASMCs) and its corresponding quantification (*n* = 3 per group). Data are presented as mean ± standard deviation (SD) from at least three independent experiments. *p < 0.05, **p < 0.01, ***p < 0.001. FGF23, fibroblast growth factor 23; GAPDH, Glyceraldehyde-3-Phosphate Dehydrogenase.

In animal experiments, the protein expression levels of FGF23 was significantly elevated in the calcification group compared with the control group (*p* < 0.05), whereas Evolocumab treatment markedly reduced their levels in the calcification + Evolocumab group (*p* < 0.05) (Figure 6A). Consistent results were observed in cellular experiments (Figure 6B), where FGF23 expression was significantly upregulated in calcified HASMCs (*p* < 0.001) and significantly downregulated following siRNA-mediated PCSK9 knockdown (*p* < 0.05). These findings suggest that FGF23 may act as mediators in the attenuation of calcification induced by PCSK9 inhibition, providing mechanistic insight into the molecular pathways underlying vascular calcification.

## Discussion

MR is a robust analytical approach that leverages genetic variation as instrumental variables to assess causal relationships between exposures and outcomes. Compared with traditional observational studies, MR effectively controls for residual and unmeasured confounders and reduces bias from reverse causality. In this study, we applied MR to provide genetic evidence for a significant association between ApoB, LDL-C, and CAC, consistent with previous clinical observations.

LDL-C is closely correlated with ApoB, with both serving as key indicators of atherogenic lipoproteins. However, accumulating evidence indicates that ApoB has superior predictive value for major adverse cardiovascular events (MACEs) compared with LDL-C (16). Johannesen et al. included 53,484 statin-free women and 41,624 statin-free men from the Copenhagen General Population Study and introduced the concept of “Excess ApoB” defined as the difference between measured ApoB and the expected ApoB predicted solely from LDL-C. Their results showed that Excess ApoB was associated with a dose-dependent increase in myocardial infarction (MI) and ASCVD risk in both sexes, and these associations remained stable across the full range of LDL-C levels (17). This suggests that ApoB provides additional predictive information beyond LDL-C alone.

Regarding CAC, ApoB also demonstrates superior clinical utility (18). Chan-Won Kim et al. conducted a multivariable-adjusted analysis comparing CAC progression among three groups: low ApoB/low LDL-C (reference), high ApoB/low LDL-C (discordant), and high ApoB/high LDL-C (concordant). Both the discordant and concordant high-ApoB groups exhibited significantly higher CAC scores compared with the reference group, and these associations remained robust after adjustment for insulin resistance and subclinical inflammation (19). Similarly, Ocampo-Torres et al. observed that ApoB was associated with CAC progression in patients with systemic lupus erythematosus (20).

Notably, our findings differ from some previous MR studies. For instance, Zhang et al. reported that while ApoB and LDL-C were causally associated with CAC in TSMR, MVMR analysis did not reveal a significant association for ApoB (21). We hypothesize that this discrepancy stems from multicollinearity between ApoB and LDL-C, as their high correlation reduces the effectiveness of the instrumental variables when included simultaneously in MVMR. Consistent with this, in our study, including both ApoB and LDL-C in the MVMR analysis markedly decreased the *F*-statistics of the instruments, with some values falling below 10. In contrast, analyzing the two variables separately substantially increased the *F*-statistics and improved the overall statistical power, supporting the robustness of our results.

At the cellular level, studies have consistently demonstrated that PCSK9 plays a role in promoting vascular calcification (22). However, clinical evidence regarding the effect of PCSK9 inhibitors on CAC is heterogeneous. Gao et al. investigated patients with moderate coronary artery disease and elevated LDL-C who received alirocumab in addition to statin therapy. After a mean follow-up of 15 months, the progression rate of CAC in the combined therapy group was significantly lower than that in the statin-only group (0.6 ± 2.2% vs. 2.7 ± 2.3%, *p* = 0.002) (6). In contrast, Nicholls et al. studied 968 patients with coronary heart disease receiving statin therapy and found that although Evolocumab further reduced LDL-C (33.5 mg/dL vs. 89.9 mg/dL, *p* < 0.0001) and promoted plaque regression (percentage change in plaque volume: −1.2% vs. +0.17%, *p* < 0.0001), there was no significant difference in coronary calcium content between the two groups after 76 weeks (1.0 ± 0.3 mm^3^ vs. 0.6 ± 0.3 mm^3^, *p* = 0.49) (7). A recent prospective imaging study using combined computed tomography angiography (CTA) and 18F-sodium fluoride (18F-NaF) positron emission tomography (PET) showed that, during a mean follow-up of 18 months, Evolocumab did not significantly alter total plaque volume but markedly reduced non-calcified plaques (from 607.3 ± 346.8 µL to 562.1 ± 337.3 µL, *p* < 0.001) and increased calcified plaques (from 108.9 ± 133.7 µL to 148.7 ± 175.3 µL, *p* < 0.001). In addition, coronary microcalcification activity decreased significantly (from 1.35 ± 1.68 to 1.08 ± 1.37, *p* = 0.004) (8).This heterogeneity may stem from four key interrelated factors: differential assessment techniques, varied study design and populations, dual interpretations of calcification's clinical value, and undefined drug mechanisms. Technically, Gao et al. (6) quantified global CAC burden via CTA-Agatston scoring, while Nicholls et al. (7) used echo-artifact-limited IVUS virtual histology to assess local plaque calcification proportion; Han et al. (8) instead focused on plaque remodeling and microcalcification activity with PET/CTA multimodal imaging, leading to apparent contradictory findings. Further variability derives from differing primary endpoints (CAC progression, plaque regression, phenotypic remodeling), diverse baseline patient characteristics (high CAC burden, predominant non-calcified plaques, statin-treated cohorts), and inconsistent PCSK9 inhibitor/statin regimens. A key ambiguity lies in calcification's clinical significance: Gao et al. framed reduced CAC progression as a direct anti-calcific benefit, whereas Nicholls et al. and Han et al. interpreted mild calcified plaque increases as a marker of plaque stabilization—supported by the latter's concurrent reduction in microcalcification activity. Finally, mechanism uncertainty persists: Nicholls et al. linked effects solely to LDL-C lowering, Gao et al. highlighted a potential association with Lp(a) reduction, and direct lipid-independent anti-calcific effects of PCSK9 inhibitors—evidenced *in vitro*—remain unconfirmed clinically.

To address this heterogeneity and clarify the potential value of PCSK9-targeted interventions for CAC, we adopted a genetic association approach to systematically evaluate the feasibility of the PCSK9 gene as a therapeutic target. Using LDL-C and ApoB as core anchors, we constructed genetic IVs strongly associated with PCSK9 expression or function within a 1 Mb region upstream and downstream of the gene, based on GWAS data. Association analyses between these IVs and CAC revealed significant positive relationships regardless of whether LDL-C or ApoB was used as the anchor. Colocalization analyses further confirmed these findings, supporting a potential causal association between PCSK9 and CAC. Complementary animal and cell experiments provided additional mechanistic and *in vivo* evidence, collectively validating the scientific rationale and translational potential of targeting PCSK9 for CAC intervention.

After confirming the association between the PCSK9 gene and CAC, we further explored the potential role of circulating inflammatory factors in mediating this relationship. Previous studies have demonstrated that the choice of *p*-value thresholds for extracting IVs can significantly affect the results. To enhance the robustness of the association, reduce heterogeneity, and improve accuracy, a *p*-value threshold of 5e-8 is commonly used (23, 24). Accordingly, In this study, we applied a threshold of 5e-8 to extract instrumental variables (IVs) for inflammatory proteins. Under this criterion, a total of 7 circulating inflammatory proteins were identified as being associated with coronary artery calcium (CAC).

In the subsequent mediation analysis, we confirmed that FGF23 is not only a risk factor for CAC but also plays a key mediating role in the pathological processes underlying PCSK9-induced CAC progression. However, caution is warranted when interpreting this finding: the extremely small *β* value in the MR analysis (Figure 4) indicates that the magnitude of this causal effect is modest at the population level. Although large-sample GWAS datasets provide sufficient statistical power to detect this subtle association, statistical significance does not equate to strong clinical relevance in individual cases. Nevertheless, the consistency of this association across multiple sensitivity analyses and its validation in experimental models support its biological plausibility, rather than a spurious correlation induced by confounding factors. Notably, the evidence supporting the role of FGF23 in vascular calcification is relatively well-established. As a cytokine primarily secreted by osteoblasts and osteocytes, FGF23 plays a central role in regulating phosphate metabolism and maintaining vitamin D homeostasis, making it a key molecule for mineral balance (25). Clinically, numerous high-quality studies have consistently confirmed the predictive value of FGF23: it is not only a strong predictor of adverse cardiovascular outcomes such as heart failure and left ventricular hypertrophy, as well as valvular calcification like aortic valve calcification (26–28), but is also closely associated with vascular wall calcification. Specifically, patients with carotid artery calcification and coronary artery calcification exhibit significantly elevated serum FGF23 levels, which correlate with disease severity (29, 30). Our mediation MR analysis further confirmed that FGF23 is a risk factor for CAC and plays a key mediating role in the pathological process through which PCSK9 promotes CAC. Importantly, these findings have been validated in both animal and *in vitro* experiments, providing multi-level evidence—from population studies to molecular mechanisms—for its role in CAC.

Building on these mechanistic insights, the integrated genetic MR and experimental findings highlight the translational potential of targeting the PCSK9-FGF23 axis for coronary artery calcification (CAC) management. First, our identification of a causal link between PCSK9-eQTL and elevated FGF23 levels (OR = 1.07, 95% CI: 1.03–1.11, *p* < 0.001) reveals that PCSK9 inhibition may exert anti-calcification effects beyond lipid lowering, by attenuating FGF23-mediated pro-calcification pathways. This supports the design of clinical trials evaluating PCSK9 inhibitors for CAC regression, with serial FGF23 measurements as a key secondary endpoint. Second, the PCSK9-FGF23 axis provides a novel basis for biomarker development: combined detection of PCSK9 and FGF23 could serve as a robust predictive panel for identifying subclinical CAC high-risk individuals, enabling personalized risk stratification. Third, our findings lay the groundwork for exploring combination therapies targeting PCSK9 and FGF23 signaling, which may yield synergistic anti-calcification effects warranting preclinical validation. Collectively, these insights bridge bench discoveries to clinical practice, positioning the PCSK9-FGF23 axis as a promising target for combating cardiovascular calcific disease.

However, several limitations should be considered when interpreting the results of this study. First, the results of this study mainly rely on data from European populations, which limits the generalizability of its conclusions. Compared with South Asian populations, PCSK9 exhibits a stronger correlation with LDL-C in Europeans (31); thus, the pathological progression rate of coronary artery calcification (CAC) may also be regulated by race-specific genes. Second, during the screening of instrumental variables for inflammatory cytokines, relaxing the threshold may introduce more “weak instrumental variables” or SNPs with horizontal pleiotropy. Even though we employed MR-PRESSO and MR-Egger tests, it is still impossible to completely rule out the risk that these SNPs affect CAC through other pathways, thereby reducing the reliability of the mediating effect of inflammatory cytokines in the mediation analysis. Third, siRNA-mediated PCSK9 knockdown and monoclonal antibody (e.g., evolocumab)-mediated PCSK9 neutralization differ in their mechanisms of action: the former inhibits intracellular PCSK9 gene expression, affecting intracellular signaling and local secretion; the latter neutralizes extracellular PCSK9 in the circulation, blocking the PCSK9–LDLR interaction without directly inhibiting intracellular synthesis or transcriptional regulation. Consequently, the two interventions exert partially overlapping but not identical biological effects in downstream pathways, particularly those related to vascular calcification and inflammation. Existing studies have indicated that intracellular and extracellular PCSK9 may possess partially distinct biological functions (32, 33). Fourth, both *in vitro* and *in vivo* experiments relied on short-term calcification models, and the MR analysis was predicated on cross-sectional GWAS datasets. Given that CAC progression in humans is a chronic process spanning years to decades, neither the short-term effects of PCSK9 inhibition observed in experimental models nor the static genetic causal estimates generated by MR analysis are able to capture the dynamic temporal fluctuations in PCSK9 expression, inflammatory cytokine profiles, and CAC burden over time. Meanwhile, the relatively limited sample size of the included datasets may reduce the statistical power of subgroup analyses, potentially hindering the detection of subtle yet biologically meaningful associations in specific subpopulations. Accordingly, long-term prospective cohort studies are planned to dynamically monitor serial CAC scores, PCSK9 expression levels, lipid profiles, and inflammatory cytokine concentrations in participants. Such longitudinal investigations will help clarify the temporal causal relationship between the PCSK9 pathway and CAC progression, and further validate the inferences derived from the MR analysis of the present study.

In summary, our study demonstrates that inhibition of PCSK9 can effectively slow the progression of coronary artery calcification, with inflammatory cytokines such as FGF23 playing key regulatory roles in this process. These findings not only provide a rationale for potentially expanding the therapeutic indications of PCSK9 inhibitors to include the attenuation of coronary artery calcification, but also suggest a novel intervention strategy targeting relevant inflammatory pathways. However, the translational and clinical value of these findings requires further validation through additional clinical studies and mechanistic investigations.

## Data Availability

The original contributions presented in the study are included in the article/Supplementary Material, further inquiries can be directed to the corresponding authors.

## References

[1] Budoff MJ Kinninger A Gransar H Achenbach S Al-Mallah M Bax JJ When does a calcium score equate to secondary prevention?: insights from the multinational CONFIRM registry. JACC Cardiovasc Imaging. (2023) 16(9):1181–9. 10.1016/j.jcmg.2023.03.008 37227328

[2] Onnis C Virmani R Kawai K Nardi V Lerman A Cademartiri F Coronary artery calcification: current concepts and clinical implications. Circulation. (2024) 149(3):251–66. 10.1161/CIRCULATIONAHA.123.065657 38227718 PMC10794033

[3] Jackson CL Garg PK Guan W Tsai MY Criqui MH Tsimikas S Lipoprotein(a) and coronary artery calcium in comparison with other lipid biomarkers: the multi-ethnic study of atherosclerosis. J Clin Lipidol. (2023) 17(4):538–48. 10.1016/j.jacl.2023.06.002 37357049 PMC10691212

[4] Masrouri S Tamehri Zadeh SS Shapiro MD Khalili D Hadaegh F. Impact of optimal cholesterol levels on subclinical atherosclerosis in the absence of risk factors in young adults. Atherosclerosis. (2024) 393:117520. 10.1016/j.atherosclerosis.2024.117520 38616451

[5] Marfella R Prattichizzo F Sardu C Paolisso P D’Onofrio N Scisciola L Evidence of an anti-inflammatory effect of PCSK9 inhibitors within the human atherosclerotic plaque. Atherosclerosis. (2023) 378:117180. 10.1016/j.atherosclerosis.2023.06.971 37422356

[6] Gao F Li YP Ma XT Wang ZJ Shi DM Zhou YJ. Effect of alirocumab on coronary calcification in patients with coronary artery disease. Front Cardiovasc Med. (2022) 9:907662. 10.3389/fcvm.2022.907662 35600486 PMC9120536

[7] Nicholls SJ Puri R Anderson T Ballantyne CM Cho L Kastelein JJP Effect of evolocumab on coronary plaque composition. J Am Coll Cardiol. (2018) 72(17):2012–21. 10.1016/j.jacc.2018.06.078 30336824

[8] Han D Tzolos E Park R Gransar H Hyun M Friedman JD Effects of evolocumab on coronary plaque composition and microcalcification activity by coronary PET and CT angiography. JACC Cardiovasc Imaging. (2025) 18(5):589–99. 10.1016/j.jcmg.2025.01.005 40178463 PMC12058403

[9] Burgess S Foley CN Zuber V. Inferring causal relationships between risk factors and outcomes from genome-wide association study data. Annu Rev Genomics Hum Genet. (2018) 19:303–27. 10.1146/annurev-genom-083117-021731 29709202 PMC6481551

[10] Davey Smith G Hemani G. Mendelian Randomization: genetic anchors for causal inference in epidemiological studies. Hum Mol Genet. (2014) 23(R1):R89–98. 10.1093/hmg/ddu328 25064373 PMC4170722

[11] Richardson TG Sanderson E Palmer TM Ala-Korpela M Ference BA Davey Smith G Evaluating the relationship between circulating lipoprotein lipids and apolipoproteins with risk of coronary heart disease: a multivariable Mendelian randomisation analysis. PLoS Med. (2020) 17(3):e1003062. 10.1371/journal.pmed.1003062 32203549 PMC7089422

[12] Zhao JH Stacey D Eriksson N Macdonald-Dunlop E Hedman ÅK Kalnapenkis A Genetics of circulating inflammatory proteins identifies drivers of immune-mediated disease risk and therapeutic targets. Nat Immunol. (2023) 24(9):1540–51. 10.1038/s41590-023-01588-w 37563310 PMC10457199

[13] Kavousi M Bos MM Barnes HJ Lino Cardenas CL Wong D Lu H Multi-ancestry genome-wide study identifies effector genes and druggable pathways for coronary artery calcification. Nat Genet. (2023) 55(10):1651–64. 10.1038/s41588-023-01518-4 37770635 PMC10601987

[14] Gummesson A Lundmark P Chen QS Björnson E Dekkers KF Hammar U A genome-wide association study of imaging-defined atherosclerosis. Nat Commun. (2025) 16(1):2266. 10.1038/s41467-025-57457-7 40164586 PMC11958696

[15] Ding R Zou X Qin Y Gong L Chen H Ma X xQTLbiolinks: a comprehensive and scalable tool for integrative analysis of molecular QTLs. Brief Bioinform. (2023) 25(1):bbad440. 10.1093/bib/bbad440 38058186 PMC10701093

[16] De Oliveira-Gomes D Joshi PH Peterson ED Rohatgi A Khera A Navar AM. Apolipoprotein B: bridging the gap between evidence and clinical practice. Circulation. (2024) 150(1):62–79. 10.1161/CIRCULATIONAHA.124.068885 38950110 PMC11219008

[17] Johannesen CDL Langsted A Nordestgaard BG Mortensen MB. Excess apolipoprotein B and cardiovascular risk in women and men. J Am Coll Cardiol. (2024) 83(23):2262–73. 10.1016/j.jacc.2024.03.423 38839200

[18] Wilkins JT Li RC Sniderman A Chan C Lloyd-Jones DM. Discordance between apolipoprotein B and LDL-cholesterol in young adults predicts coronary artery calcification: the CARDIA study. J Am Coll Cardiol. (2016) 67(2):193–201. 10.1016/j.jacc.2015.10.055 26791067 PMC6613392

[19] Kim C-W Hong S Chang Y Lee JA Shin H Ryu S. Discordance between apolipoprotein B and low-density lipoprotein cholesterol and progression of coronary artery calcification in middle age. Circ J. (2021) 85(6):900–7. 10.1253/circj.CJ-20-0692 33311006

[20] Ocampo-Torres MC Hernández-Molina G Criales-Vera S Sánchez-Guerrero J Lara-Reyes P Romero-Díaz J. Coronary artery calcification progression in patients with systemic lupus erythematosus. J Rheumatol. (2024) 51(10):991–6. 10.3899/jrheum.2024-0040 38950947

[21] Zhang P Wang W Xu Q Cui J Zhu M Li Y Genetic association of circulating lipids and lipid-lowering drug targets with vascular calcification. Atherosclerosis. (2025) 403:119136. 10.1016/j.atherosclerosis.2025.119136 39985880

[22] Lupo MG Bressan A Donato M Canzano P Camera M Poggio P PCSK9 Promotes arterial medial calcification. Atherosclerosis. (2022) 346:86–97. 10.1016/j.atherosclerosis.2022.01.015 35135698

[23] Bottigliengo D Foco L Seibler P Klein C König IR Del Greco MF. A Mendelian randomization study investigating the causal role of inflammation on Parkinson’s disease. Brain J Neurol. (2022) 145(10):3444–53. 10.1093/brain/awac193 PMC9586538 35656776

[24] Ren F Jin Q Jin Q Qian Y Ren X Liu T Genetic evidence supporting the causal role of gut microbiota in chronic kidney disease and chronic systemic inflammation in CKD: a bilateral two-sample Mendelian randomization study. Front Immunol. (2023) 14:1287698. 10.3389/fimmu.2023.1287698 38022507 PMC10652796

[25] Morita H Hoshiga M. Fibroblast growth factors in cardiovascular disease. J Atheroscler Thromb. (2024) 31(11):1496–511. 10.5551/jat.RV22025 39168622 PMC11537794

[26] Patel RB Ning H de Boer IH Kestenbaum B Lima JAC Mehta R Fibroblast growth factor 23 and long-term cardiac function: the multi-ethnic study of atherosclerosis. Circ Cardiovasc Imaging. (2020) 13(11):e011925. 10.1161/CIRCIMAGING.120.011925 33161733 PMC7665116

[27] Ter Maaten JM Voors AA Damman K van der Meer P Anker SD Cleland JG Fibroblast growth factor 23 is related to profiles indicating volume overload, poor therapy optimization and prognosis in patients with new-onset and worsening heart failure. Int J Cardiol. (2018) 253:84–90. 10.1016/j.ijcard.2017.10.010 29306478

[28] Morita H Takeda Y Fujita S Okamoto Y Sakane K Teramoto K Gender specific association between Serum fibroblast growth factor 23/α-klotho and coronary artery and aortic valve calcification. J Atheroscler Thromb. (2015) 22(12):1338–46. 10.5551/jat.30635 26279337

[29] Yamada S Giachelli CM. Vascular calcification in CKD-MBD: roles for phosphate, FGF23, and klotho. Bone. (2017) 100:87–93. 10.1016/j.bone.2016.11.012 27847254 PMC5429216

[30] Kumar T Mohanty S Rani A Malik A Kumar R Bhashker G. Fibroblast growth factor-23 in Pre-dialysis chronic kidney disease patients and its correlation with carotid artery calcification. Indian J Nephrol. (2022) 32(6):560–6. 10.4103/ijn.IJN_506_20 36704592 PMC9872934

[31] Wu S Meena D Smith A Huang J Otto GW Ko Y-H Identification of protein targets for dyslipidaemia and cardiovascular diseases among people with south Asian ancestry: a Mendelian randomisation study. Lancet Reg Health Southeast Asia. (2025) 39:100621. 10.1016/j.lansea.2025.100621 40689090 PMC12271078

[32] Tang Y Li S-L Hu J-H Sun K-J Liu L-L Xu D-Y. Research progress on alternative non-classical mechanisms of PCSK9 in atherosclerosis in patients with and without diabetes. Cardiovasc Diabetol. (2020) 19(1):33. 10.1186/s12933-020-01009-4 32169071 PMC7071562

[33] Roth EM Davidson MH. PCSK9 Inhibitors: mechanism of action, efficacy, and safety. Rev Cardiovasc Med. (2018) 19(S1):S31–46. 10.3909/ricm19S1S0002 30207556

