# Supplementary material for: Genetic association between PCSK9 and coronary artery calcification mediated by inflammatory cytokines
Source: Front Cardiovasc Med. 2026 Feb 16;13:1767013. doi: 10.3389/fcvm.2026.1767013 (PMC12950672; doi:10.3389/fcvm.2026.1767013)
Supplement: Supplementary file 1 [file Datasheet1.docx]

***Supplementary Material***

Supplementary table1:

**STROBE-MR checklist of recommended items to address in reports of Mendelian randomization studies**^1^ ^2^

| **Item No.** | **Section** | **Checklist item** | **Page No.** | **Relevant text from manuscript** |
| --- | --- | --- | --- | --- |
| 1 | **TITLE and ABSTRACT** | Indicate Mendelian randomization (MR) as the study’s design in the title and/or the abstract if that is a main purpose of the study | 1-2 | Abstract(methods): First, we used two-sample Mendelian randomization (MR) and multivariable Mendelian randomization to identify lipid profiles genetically associated with coronary artery calcification. Subsequently, we investigated the value of the PCSK9 gene as a potential therapeutic target for CAC through drug target MR and colocalization analysis, and screened for potential inflammatory mediators via Mediation MR analyses. |
|  | **INTRODUCTION** |  |  |  |
| 2 | **Background** | Explain the scientific background and rationale for the reported study. What is the exposure? Is a potential causal relationship between exposure and outcome plausible? Justify why MR is a helpful method to address the study question | 2-3 | Coronary artery calcification (CAC), a hallmark of coronary atherosclerosis, links closely to dysregulated lipid metabolism and chronic inflammation. Proprotein convertase subtilisin/kexin type 9 (PCSK9) inhibitors exert potent lipid-lowering and anti-inflammatory effects, holding translational potential for vascular calcification intervention. However, evidence on the impact of PCSK9 inhibition on vascular calcification remains inconsistent in observational studies. These studies are inherently susceptible to residual confounding (e.g., lifestyle factors) and reverse causation (i.e., advanced vascular calcification may upregulate PCSK9 expression), which preclude definitive causal inference. In this study, the exposure was defined as genetically predicted PCSK9 levels, and the outcome was CAC. A potential causal relationship between PCSK9 and CAC is biologically plausible, given that PCSK9-mediated alterations in lipid metabolism and inflammatory responses directly regulate the pathological processes of atherosclerotic calcification. |
| 3 | **Objectives** | State specific objectives clearly, including pre-specified causal hypotheses (if any). State that MR is a method that, under specific assumptions, intends to estimate causal effects | 3-4 | The primary objectives of this study were to: 1) examine the potential causal relationships between five common lipid traits, inflammatory cytokines, and coronary artery calcification (CAC) using a two-sample Mendelian randomization (MR) design; 2) identify independent lipid-related exposures associated with CAC via multivariable MR; 3) evaluate the therapeutic relevance of the PCSK9 gene in CAC through drug-target MR and colocalization analyses; and 4) explore potential inflammatory mediators linking PCSK9 to CAC by mediation MR analyses. We pre-specified the causal hypothesis that genetically predicted PCSK9 levels exert a causal effect on CAC progression, which may be mediated by lipid metabolism dysregulation and altered inflammatory cytokine levels. Mendelian randomization is a method that, under specific assumptions (instrument relevance, independence from confounders, and exclusion restriction), intends to estimate the causal effects between exposures and outcomes while mitigating unmeasured confounding and reverse causality. |
|  | **METHODS** |  |  |  |
| 4 | **Study design and data sources** | Present key elements of the study design early in the article. Consider including a table listing sources of data for all phases of the study. For each data source contributing to the analysis, describe the following: |  |  |
|  | a) | Setting: Describe the study design and the underlying population, if possible. Describe the setting, locations, and relevant dates, including periods of recruitment, exposure, follow-up, and data collection, when available. | 4 | Genome-wide association study (GWAS) data for lipid traits were obtained from the IEU OpenGWAS database, including apolipoprotein A1 (ApoA1), ApoB, high-density lipoprotein cholesterol (HDL-C), low-density lipoprotein cholesterol (LDL-C), and triglycerides (TG), with corresponding GWAS IDs ieu-b-107 to ieu-b-111. Data on circulating inflammation-related proteins were derived from a meta-analysis of 91 inflammatory proteins across 11 cohorts, comprising 14,824 participants of European ancestry, and their full GWAS summary statistics are available in the GWAS Catalog under accession numbers GCST90274758 to GCST90274848. Summary statistics for coronary artery calcification (CAC) were retrieved from a multi-ancestry GWAS meta-analysis; to minimize population stratification bias, only data from participants of European ancestry were used (GWAS Catalog accession number: GCST90278456). An additional CAC dataset (GCST90503074) was employed for replication analyses. PCSK9 expression quantitative trait locus (eQTL) data extracted from the GTEx V8 database were used as the exposure. |
|  | b) | Participants: Give the eligibility criteria, and the sources and methods of selection of participants. Report the sample size, and whether any power or sample size calculations were carried out prior to the main analysis |  | A complete summary of all GWAS datasets used in this study is provided in Table 1. |
|  | c) | Describe measurement, quality control and selection of genetic variants | 4-5 | In the MR analysis, single nucleotide polymorphisms (SNPs) strongly associated with the exposures were selected as IVs. For the five lipid traits and 91 inflammatory traits, SNPs were identified using a genome-wide significance threshold of p < 5 × 10⁻⁸. To ensure independence, SNPs in linkage disequilibrium (LD) were excluded using a clumping threshold of 10,000 kb and r² < 0.001.The strength of each IV was assessed using the F-statistic, calculated as: F = R² (N – 2) / (1 – R²), where R² = 2β² × EAF × (1 – EAF), with β representing the SNP effect size on the exposure and EAF the effect allele frequency. An F-statistic > 10 was considered indicative of sufficient instrument strength. SNPs failing to meet this criterion were excluded, and no proxy SNPs were used. The Steiger test was performed to confirm the causal direction, thereby minimizing bias from reverse causality. For drug-target MR analyses, SNPs located within 1 Mb upstream and downstream of the PCSK9 locus were extracted from GWAS datasets of lipid traits. Here, a clumping threshold of 100 kb and r² < 0.3 was applied. In addition, PCSK9-eQTL data were obtained from the GTEx V8 database, restricted to the “Whole Blood” tissue type, with a stringent threshold of p < 5 × 10⁻⁸ for SNP selection. |
|  | d) | For each exposure, outcome, and other relevant variables, describe methods of assessment and diagnostic criteria for diseases |  | The exposures in this study include lipid traits (LDL-C, HDL-C, triglycerides, and apolipoproteins), inflammatory cytokines, and PCSK9 expression-associated eQTLs. Lipid traits were measured in the UK Biobank using standardized enzymatic assays; inflammatory cytokines were quantified in plasma using the Olink Target Inflammation panel (proximity extension assay); and PCSK9-eQTLs were derived from cis-eQTL data for liver tissue in individuals of European ancestry from the GTEx v8 database.The outcome was coronary artery calcium (CAC), quantified by Agatston scores from cardiac computed tomography. GWAS summary statistics for CAC were obtained from a multi-ancestry meta-analysis, but only results from participants of European ancestry were used to minimize population stratification bias (see Reference 13).No clinical disease diagnoses were used as outcomes; therefore, formal diagnostic criteria for diseases are not applicable. Covariates such as age and sex were sourced from baseline records in the respective datasets. |
|  | e) | Provide details of ethics committee approval and participant informed consent, if relevant |  | This study utilized publicly available genome-wide association study (GWAS) summary data, which have been previously approved by the respective ethics committees of the original studies and obtained written informed consent from all participants. Detailed information on ethics approval and informed consent can be found in the original publications of the GWAS datasets. |
| 5 | **Assumptions** | Explicitly state the three core IV assumptions for the main analysis (relevance, independence and exclusion restriction) as well assumptions for any additional or sensitivity analysis | 3-4 | This study strictly adhered to the three core assumptions of MR. First, the relevance assumption requires that selected genetic variants are strongly associated with the exposure, thereby serving as valid proxies. Second, the independence assumption states that, conditional on the exposure, the genetic variants remain independent of confounders affecting the outcome. Third, the exclusion restriction assumption requires that genetic variants influence the outcome only through the exposure, without direct or exposure-independent indirect pathways.For sensitivity analyses, we performed MR-Egger regression and leave-one-out analysis. For MR-Egger regression, we assumed that the horizontal pleiotropy effects were balanced, which allowed for the estimation of causal effects even in the presence of unbalanced pleiotropy to a certain extent. The leave-one-out analysis was conducted without additional assumptions beyond the three core MR assumptions; its purpose was to assess whether the overall causal estimate was driven by a single influential genetic variant, thereby verifying the robustness of the main findings. |
| 6 | **Statistical methods: main analysis** | Describe statistical methods and statistics used |  |  |
|  | a) | Describe how quantitative variables were handled in the analyses (i.e., scale, units, model) |  | All quantitative variables involved in the analyses were treated as continuous variables. Specifically, genetically predicted levels of PCSK9, five common lipid traits (e.g., LDL-C, HDL-C, triglycerides) and inflammatory cytokines were standardized as z-scores to ensure comparability across different exposure variables, with original units following the respective GWAS summary data (e.g., mmol/L for lipid traits). For the outcome variable, coronary artery calcification (CAC) was quantified as a continuous CAC score, consistent with the measurement scale in the original GWAS dataset. The primary MR analysis was conducted using the inverse-variance weighted (IVW) model, which efficiently synthesizes causal estimates from multiple genetic instruments; sensitivity analyses adopted the weighted median model and MR-Egger regression to account for potential heterogeneity and pleiotropy. |
|  | b) | Describe how genetic variants were handled in the analyses and, if applicable, how their weights were selected | 4-5 | Strongly exposure-associated SNPs were selected as IVs using p < 5×10⁻⁸. LD SNPs were excluded via clumping (10,000 kb, r² < 0.001). IV strength was assessed by F-statistic (F > 10), with formula F = R² (N – 2) / (1 – R²)(R² = 2β² × EAF × (1 – EAF)); weak IVs were excluded without proxy SNPs. Steiger tests confirmed causal direction. For drug-target MR, PCSK9 locus ±1 Mb SNPs were extracted (clumping: 100 kb, r² < 0.3). PCSK9-eQTL data were from GTEx V8 (Whole Blood, p < 5×10⁻⁸). |
|  | c) | Describe the MR estimator (e.g. two-stage least squares, Wald ratio) and related statistics. Detail the included covariates and, in case of two-sample MR, whether the same covariate set was used for adjustment in the two samples | 5-6 | MR analyses were performed using multiple approaches, with inverse variance weighting (IVW) as the primary method. IVW yields robust causal estimates under the assumption of all valid genetic instruments and accommodates SNP heterogeneity. Cochran’s Q test assessed heterogeneity (p < 0.05 = significant heterogeneity, indicating fixed-effects model inadequacy). Outlier detection was conducted via MR-PRESSO; corrected causal estimates were derived using fixed-effects IVW after outlier removal. MR-Egger intercept evaluated horizontal pleiotropy, with the caveat of its reduced statistical power. |
|  | d) | Explain how missing data were addressed |  | No missing data were present in the publicly available GWAS summary datasets used for MR analyses. All genetic variants and their corresponding effect estimates, allele frequencies, and p-values were complete for the selected exposures and outcomes. |
|  | e) | If applicable, indicate how multiple testing was addressed | 3-4 | Given that the causal inferences derived from MR analyses were further validated by subsequent in vivo animal experiments and in vitro cell experiments, no formal multiple testing correction was applied to the MR results. A nominal significance level of p < 0.05 was used to identify potential causal associations worthy of follow-up experimental validation. |
| 7 | **Assessment of assumptions** | Describe any methods or prior knowledge used to assess the assumptions or justify their validity | 3-4 | The validity of the three core instrumental variable (IV) assumptions for MR analyses was assessed using a combination of statistical methods and domain prior knowledge.  1.Relevance assumption: Confirmed by selecting SNPs with genome-wide significance (p < 5×10⁻⁸) for exposures and evaluating instrument strength via F-statistic (F > 10), consistent with the widely accepted threshold in MR studies to exclude weak instruments.  2.Independence assumption: Justified by two strategies: (1) excluding SNPs in linkage disequilibrium (LD, clumping threshold: 10,000 kb, r² < 0.001) to ensure IV independence; (2) relying on the biological principle that germline genetic variants are randomly assigned at conception and thus not associated with confounding factors of the exposure-outcome relationship.  3.Exclusion restriction assumption: Validated using multiple sensitivity analyses: (1) MR-Egger regression to detect and adjust for directional horizontal pleiotropy, with the intercept term indicating pleiotropic effects; (2) MR-PRESSO test to identify and remove outlier SNPs that might violate the assumption; (3) leave-one-out analysis to confirm that no single SNP unduly drove the causal estimate. Additionally, the Steiger test was performed to verify the causal direction, minimizing the risk of reverse causality that would otherwise violate the assumption. |
| 8 | **Sensitivity analyses and additional analyses** | Describe any sensitivity analyses or additional analyses performed (e.g. comparison of effect estimates from different approaches, independent replication, bias analytic techniques, validation of instruments, simulations) | 3-4 | Sensitivity analyses were performed to verify the robustness of causal estimates: MR-Egger regression was used to detect and adjust for directional horizontal pleiotropy; weighted median method was applied to enhance result stability when partial instruments were invalid; MR-PRESSO test identified and removed outlier SNPs to reduce bias; leave-one-out analysis assessed whether individual SNPs unduly drove the overall result. Additional analyses included F-statistic evaluation (F > 10) to validate instrument strength, Steiger test to confirm causal direction, and drug-target MR analysis focusing on PCSK9 locus (±1 Mb SNPs, clumping: 100 kb, r² < 0.3) with eQTL data from GTEx V8 (Whole Blood) for instrument validation. |
| 9 | **Software and pre-registration** |  |  |  |
|  | a) | Name statistical software and package(s), including version and settings used | 8 | Mendelian randomization analyses were conducted in R software (version 4.3.3) using the packages TwoSampleMR, MRPRESSO, and coloc. Effect sizes (ES) with 95% confidence intervals (CI) were reported as results. Visualization of findings was performed using the R packages locuscomparer and forestploter with default parameter settings unless specified otherwise. |
|  | b) | State whether the study protocol and details were pre-registered (as well as when and where) |  | This study was an exploratory analysis based on publicly available GWAS summary data, and the study protocol was not pre-registered. |
|  | **RESULTS** |  |  |  |
| 10 | **Descriptive data** |  |  |  |
|  | a) | Report the numbers of individuals at each stage of included studies and reasons for exclusion. Consider use of a flow diagram |  | Final analytical samples included 393,193–441,016 UK Biobank participants for lipid traits, 14,824 individuals from 11 cohorts with Olink inflammation proteomics, 838 GTEx v8 donors for *PCSK9* eQTLs, and 28,655 European-ancestry participants from a multi-ancestry GWAS meta-analysis of coronary artery calcium (CAC), with non-European data excluded to minimize population stratification. Detailed inclusion/exclusion criteria and quality control procedures are reported in References 11–13; however, as this study relies on summary- or consortium-level data, stage-specific participant counts and a full flow diagram are not available. |
|  | b) | Report summary statistics for phenotypic exposure(s), outcome(s), and other relevant variables (e.g. means, SDs, proportions) |  | Summary statistics for phenotypic variables are available only for the UK Biobank lipid GWAS: mean (SD) age was 56.9 (7.8) years, 54.2% of participants were women, mean LDL cholesterol was 3.57 (0.87) mmol/L, mean HDL cholesterol was 1.45 (0.38) mmol/L, and median triglycerides was 1.50 (IQR: 1.11) mmol/L. Individual-level phenotypic summary statistics (e.g., means, SDs, proportions) were not reported in the publicly available summary datasets for the inflammatory protein GWAS (Reference 12), *PCSK9* expression data from GTEx v8, or the coronary artery calcium (CAC) GWAS meta-analysis, and are therefore not presented here. |
|  | c) | If the data sources include meta-analyses of previous studies, provide the assessments of heterogeneity across these studies |  | Among the data sources, the summary statistics for coronary artery calcium (CAC) were derived from a multi-ancestry GWAS meta-analysis; however, assessments of between-study heterogeneity (e.g., I², Cochran’s Q) were not reported in the original publication or in the publicly available summary data used in this study. The other data sources—UK Biobank GWAS, multi-cohort Olink proteomics, and GTEx v8—are based on primary or consortium-level analyses, not meta-analyses of previously published studies. Therefore, heterogeneity assessments are either not applicable or not available. |
|  | d) | For two-sample MR:  i.  Provide justification of the similarity of the genetic variant-exposure associations between the exposure and outcome samples  ii.  Provide information on the number of individuals who overlap between the exposure and outcome studies |  | 1. Justification of genetic variant-exposure association similarity: Exposure and outcome samples shared the same ancestral background (European ancestry); exposure traits were quantified using standardized methods; IVs were genome-wide significant (P < 5×10⁻⁸) in both datasets, with consistent effect direction and magnitude.   ii. Sample overlap information: No overlapping individuals between exposure and outcome studies (datasets from independent GWAS consortia, verified via unique sample identifiers). |
| 11 | **Main results** |  |  |  |
|  | a) | Report the associations between genetic variant and exposure, and between genetic variant and outcome, preferably on an interpretable scale |  | For the associations between genetic variants and exposures, as well as between genetic variants and outcomes, all estimates were quantified and reported as beta coefficients (β) with corresponding standard errors (SE) on an interpretable scale consistent with genetic association studies. For the two-sample MR analyses with coronary artery calcification (CAC) as the outcome, the estimates were as follows: ApoA1 (β=-0.054, SE=0.068), ApoB (β=0.496, SE=0.074), HDL (β=-0.248, SE=0.069), LDL (β=0.576, SE=0.086), and TG (β=0.303, SE=0.066). For the mediation MR analyses, the respective estimates were: PCSK9-eQTL and FGF23 (β=0.067, SE=0.017), FGF23 and CAC (β=0.360, SE=0.161), and PCSK9-eQTL and CAC (β=0.174, SE=0.033). |
|  | b) | Report MR estimates of the relationship between exposure and outcome, and the measures of uncertainty from the MR analysis, on an interpretable scale, such as odds ratio or relative risk per SD difference |  | For two-sample MR (IVW method), the results were as follows: ApoA1 (OR = 0.95, 95% CI: 0.83–1.08), ApoB (OR = 1.64, 95% CI: 1.42–1.90), HDL-C (OR = 0.78, 95% CI: 0.68–0.89), LDL-C (OR = 1.78, 95% CI: 1.50–2.51), and TG (OR = 1.35, 95% CI: 1.19–1.54). For multivariable MR, the results were: LDL-C (OR = 1.73, 95% CI: 1.46–2.06) and ApoB (OR = 1.57, 95% CI: 1.33–1.84).  For the mediation MR analyses, the respective estimates were OR = 1.07 (95% CI: 1.03–1.11) for PCSK9-eQTL and FGF23, OR = 1.43 (95% CI: 1.05–1.96) for FGF23 and CAC, and OR = 1.19 (95% CI: 1.11–1.27) for PCSK9-eQTL and CAC. |
|  | c) | If relevant, consider translating estimates of relative risk into absolute risk for a meaningful time period |  | Given that the present study was based on genetic association and Mendelian randomization analyses without a defined follow-up period or incident outcome data over a specific timeframe, the conversion of relative risk estimates to absolute risk values was not applicable herein. |
|  | d) | Consider plots to visualize results (e.g. forest plot, scatterplot of associations between genetic variants and outcome versus between genetic variants and exposure) |  | Relevant results were visualized using key plots consistent with Mendelian randomization analysis guidelines, including a forest plot for the overall causal effect estimates and a scatterplot of genetic variant-outcome associations versus genetic variant-exposure associations. |
| 12 | **Assessment of assumptions** |  |  |  |
|  | a) | Report the assessment of the validity of the assumptions |  | Relevance assumption: IVs genome-wide significant (P < 5×10⁻⁸) for exposures; all F-statistics > 10 (no weak instrument bias).  Independence assumption: IVs excluded if overlapping with CAC confounders per GWAS data/literature.  Exclusion restriction assumption: No significant horizontal pleiotropy (heterogeneity/pleiotropy tests); no SNP-driven bias (leave-one-out analysis); reverse causality excluded (bidirectional MR). |
|  | b) | Report any additional statistics (e.g., assessments of heterogeneity across genetic variants, such as *I^2^*, Q statistic or E-value) |  | The Q statistic for assessing heterogeneity across genetic variants in the MR analyses with coronary artery calcification (CAC) was reported, and detailed results are presented in Table 2.2. |
| 13 | **Sensitivity analyses and additional analyses** |  |  |  |
|  | a) | Report any sensitivity analyses to assess the robustness of the main results to violations of the assumptions |  | Sensitivity analyses included heterogeneity & pleiotropy testing, causal relationship direction assessment (bidirectional MR, Steiger tests and reverse MR), replication with independent dataset, MVMR stratification for collinearity, and the leave-one-out method. |
|  | b) | Report results from other sensitivity analyses or additional analyses |  | Heterogeneity and pleiotropy testing showed no significance, validating the IVW results. Reverse causality evaluation via Steiger tests and reverse MR excluded reverse causation. Replication with the independent dataset GCST90503074 yielded consistent results, confirming the robustness of primary findings. MVMR stratification for collinearity resolved collinearity issues and validated the independent effects of relevant exposures. The leave-one-out method identified no obviously abnormal SNPs. |
|  | c) | Report any assessment of direction of causal relationship (e.g., bidirectional MR) |  | To clarify the direction of causality and exclude the possibility of reverse causation, Steiger tests and reverse MR analyses were performed. The results indicated no evidence of reverse causality between ApoB, LDL-C, and CAC. |
|  | d) | When relevant, report and compare with estimates from non-MR analyses |  | No conventional observational analysis was performed in this study. However, our MR-derived findings were consistent with those of previous clinical studies: LDL and apoB were identified as risk factors for CAC; PCSK9 inhibition was shown to attenuate CAC progression, which was in line with the results reported in Reference 6; and FGF23 was found to be involved in the pathogenesis of CAC, consistent with the conclusions of References 29 and 30. These consistencies further corroborate the robustness of our causal inferences. |
|  | e) | Consider additional plots to visualize results (e.g., leave-one-out analyses) |  | The scatter plots, funnel plots, and leave-one-out sensitivity analysis plots associated with the MR analyses in this study have been included in the Supplementary Material. |
|  | **DISCUSSION** |  |  |  |
| 14 | **Key results** | Summarize key results with reference to study objectives | 8-10 | This study focused on the causal effects of lipid traits on coronary artery calcification (CAC), the therapeutic potential of PCSK9 inhibition, and the mediating role of inflammatory cytokines, with the key results summarized as follows: TSMR confirmed that ApoB, LDL-C, and TG increase the risk of CAC, while HDL-C reduces it. MVMR verified the independent causal effects of ApoB and LDL-C on CAC, and the results were validated using an independent dataset with no evidence of reverse causality. Drug-target MR and colocalization analyses indicated that PCSK9-targeted regulation can reduce CAC risk. Inflammatory mediator analysis showed that FGF23 mediates 13.86% of the effect of PCSK9 on CAC, whereas the mediating effect of IL-6 is not significant. In vivo and in vitro experiments confirmed that PCSK9 inhibition alleviates vascular calcification, and FGF23 is regulated by PCSK9 and mediates its anti-calcification effect. |
| 15 | **Limitations** | Discuss limitations of the study, taking into account the validity of the IV assumptions, other sources of potential bias, and imprecision. Discuss both direction and magnitude of any potential bias and any efforts to address them | 14-15 | 1. IV Assumption Validity & Bias: Weak instruments and horizontal pleiotropy may be introduced during inflammatory cytokine IV screening, thereby impairing the validity of IV assumptions (specifically violating the exclusivity assumption). Bias direction: overestimates or underestimates the mediation effect; magnitude correlates with the count and effect size of pleiotropic SNPs. Mitigation measures: MR-PRESSO and MR-Egger tests were applied; however, residual pleiotropy persists, reducing the reliability of the mediation effect.  2. Population Stratification Bias: European-ancestry GWAS data used; PCSK9-LDL-C correlation is ethnicity-specific (Ref. 31). Bias: limits extrapolation to non-Europeans (moderate magnitude). No targeted mitigation; multi-ethnic cohort validation required.  3. Imprecision & Experimental Design Bias: Short-term calcification models used, while human CAC is a chronic process. Imprecision: fails to reflect long-term efficacy and cytokine dynamic changes. Bias: overestimates intervention effect (considerable magnitude). No experimental design adjustments; long-term models needed for validation.  4. Additional Imprecision: European cohorts lack genetic diversity, reducing IV analysis power for non-Europeans (secondary impact). |
| 16 | **Interpretation** |  |  |  |
|  | a) | Meaning: Give a cautious overall interpretation of results in the context of their limitations and in comparison with other studies |  | Population limit: European GWAS-based PCSK9-CAC associations; ethnicity-specific genetic effects prevent non-European extrapolation, consistent with prior ethnic heterogeneity findings; multi-ethnic validation required.  Mediation restraint: Inflammatory cytokine mediation supported but cautious; MR-PRESSO/MR-Egger reduce pleiotropy bias, residual pathway interference remains; effect size may deviate (aligns with MR consensus).  Model mismatch: Short-term animal/cellular data show acute PCSK9 inhibition effects; human CAC is chronic—findings support biological plausibility, not long-term clinical efficacy (consistent with long-term intervention studies).  Overall: Valuable PCSK9-inflammation-CAC evidence, constrained by population, MR, and model limits; future multi-ethnic, long-term studies needed for validation. |
|  | b) | Mechanism: Discuss underlying biological mechanisms that could drive a potential causal relationship between the investigated exposure and the outcome, and whether the gene-environment equivalence assumption is reasonable. Use causal language carefully, clarifying that IV estimates may provide causal effects only under certain assumptions | 13-14 | PCSK9 can downregulate low-density lipoprotein receptors and elevate low-density lipoprotein cholesterol levels, induce vascular endothelial dysfunction and pro-inflammatory polarization of macrophages, stimulate the secretion of pro-inflammatory cytokines, accelerate the calcification of vascular smooth muscle cells and the formation of atherosclerotic plaques, thereby promoting the progression of coronary artery calcification; the gene-environment equivalence assumption is tentatively reasonable in that PCSK9-related genetic variants and environmental interventions (e.g., PCSK9 inhibitor therapy) both affect the risk of coronary artery calcification by regulating lipid metabolism and inflammatory pathways, but genetic variants may exert pleiotropic effects independent of canonical pathways and environmental interventions are characterized by targeting and time limitation, so the rationality of this assumption requires cautious validation; meanwhile, it should be clarified that instrumental variable estimates can only infer the potential causal effect of the PCSK9-inflammation-CAC pathway when the three core IV assumptions (relevance, independence, exclusivity) are fully satisfied, and residual pleiotropy and uncontrolled confounding factors may violate these assumptions, thus the causal interpretation of the results must be predicated on the validity of these premises. |
|  | c) | Clinical relevance: Discuss whether the results have clinical or public policy relevance, and to what extent they inform effect sizes of possible interventions | 14 | Targeting the PCSK9-FGF23 axis has translational value for coronary artery calcification (CAC) management; the identified causal link (OR = 1.07, 95% CI: 1.03–1.11) quantifies the effect of PCSK9 inhibition on FGF23-mediated calcification, providing a basis for Phase II/III clinical trials (with FGF23 as a secondary endpoint) and public policies regarding cost-effectiveness in high-risk populations. Meanwhile, combined detection of PCSK9 and FGF23 enables prediction of high-risk subclinical CAC and personalized stratification, offering insights for public policies on precision cardiovascular disease (CVD) prevention and healthcare resource allocation. Additionally, dual targeting of PCSK9 and FGF23 may enhance anti-calcification efficacy, and preclinical validation to quantify effect sizes will guide clinical trial design and treatment guideline formulation. Overall, this links mechanisms to interventions and provides core evidence for individualized clinical management and public health policy development. |
| 17 | **Generalizability** | Discuss the generalizability of the study results (a) to other populations, (b) across other exposure periods/timings, and (c) across other levels of exposure |  | (a) To other populations: Limited to non-Europeans. European-ancestry GWAS data used; PCSK9-LDL-C correlation and CAC progression are ethnicity-specific. Genetic heterogeneity forbids direct extrapolation; multi-ethnic validation needed.  (b) Across exposure periods/timings: Not applicable to long-term exposure/stages. Short-term models used; human CAC is chronic (years-decades). Only short-term effects captured; no long-term inflammatory dynamics or intervention efficacy reflected.  (c) Across exposure levels: Unconfirmed across intensities. Alternative pathways not fully excluded. No stratification by exposure severity; dose-spectrum consistency and non-linear relationships unclear. |
|  | **OTHER INFORMATION** |  |  |  |
| 18 | **Funding** | Describe sources of funding and the role of funders in the present study and, if applicable, sources of funding for the databases and original study or studies on which the present study is based | 16 | This project was supported by the Medical Scientific Research Project of Joint Logistic Support Force (No. XK20240204) and the Medical-Education Collaboration Innovation Fund of Jiangsu University (JDYY2023113). The funders had no role in the study design, data collection and analysis, decision to publish, or preparation of the manuscript. The genome-wide association study (GWAS) summary data used in this work were obtained from publicly available repositories, and the funding sources of the original GWAS projects are detailed in their respective publications (see Table 1 for references). |
| 19 | **Data and data sharing** | Provide the data used to perform all analyses or report where and how the data can be accessed, and reference these sources in the article. Provide the statistical code needed to reproduce the results in the article, or report whether the code is publicly accessible and if so, where | 15-16 | GWAS data for lipid traits are available for download from <https://gwas.mrcieu.ac.uk>. GWAS data on coronary artery calcification (CAC) can be accessed via the European Bioinformatics Institute (EBI) GWAS Catalog (<https://www.ebi.ac.uk/gwas/>), with detailed information on these data sources provided in Table 1. PCSK9-eQTL data were retrieved from the GTEx V8 database (<https://gtexportal.org/home/>).  The statistical code used to reproduce the study results is available upon reasonable request to the corresponding author. The code was written in R (version 4.3.3) with detailed annotations for each analysis step. |
| 20 | **Conflicts of Interest** | All authors should declare all potential conflicts of interest | 16 | All authors declare that they have no competing interests. |

This checklist is copyrighted by the Equator Network under the Creative Commons Attribution 3.0 Unported (CC BY 3.0) license.

1. Skrivankova VW, Richmond RC, Woolf BAR, Yarmolinsky J, Davies NM, Swanson SA, et al. Strengthening the Reporting of Observational Studies in Epidemiology using Mendelian Randomization (STROBE-MR) Statement. JAMA. 2021;under review.

2. Skrivankova VW, Richmond RC, Woolf BAR, Davies NM, Swanson SA, VanderWeele TJ, et al. Strengthening the Reporting of Observational Studies in Epidemiology using Mendelian Randomisation (STROBE-MR): Explanation and Elaboration. BMJ. 2021;375:n2233.


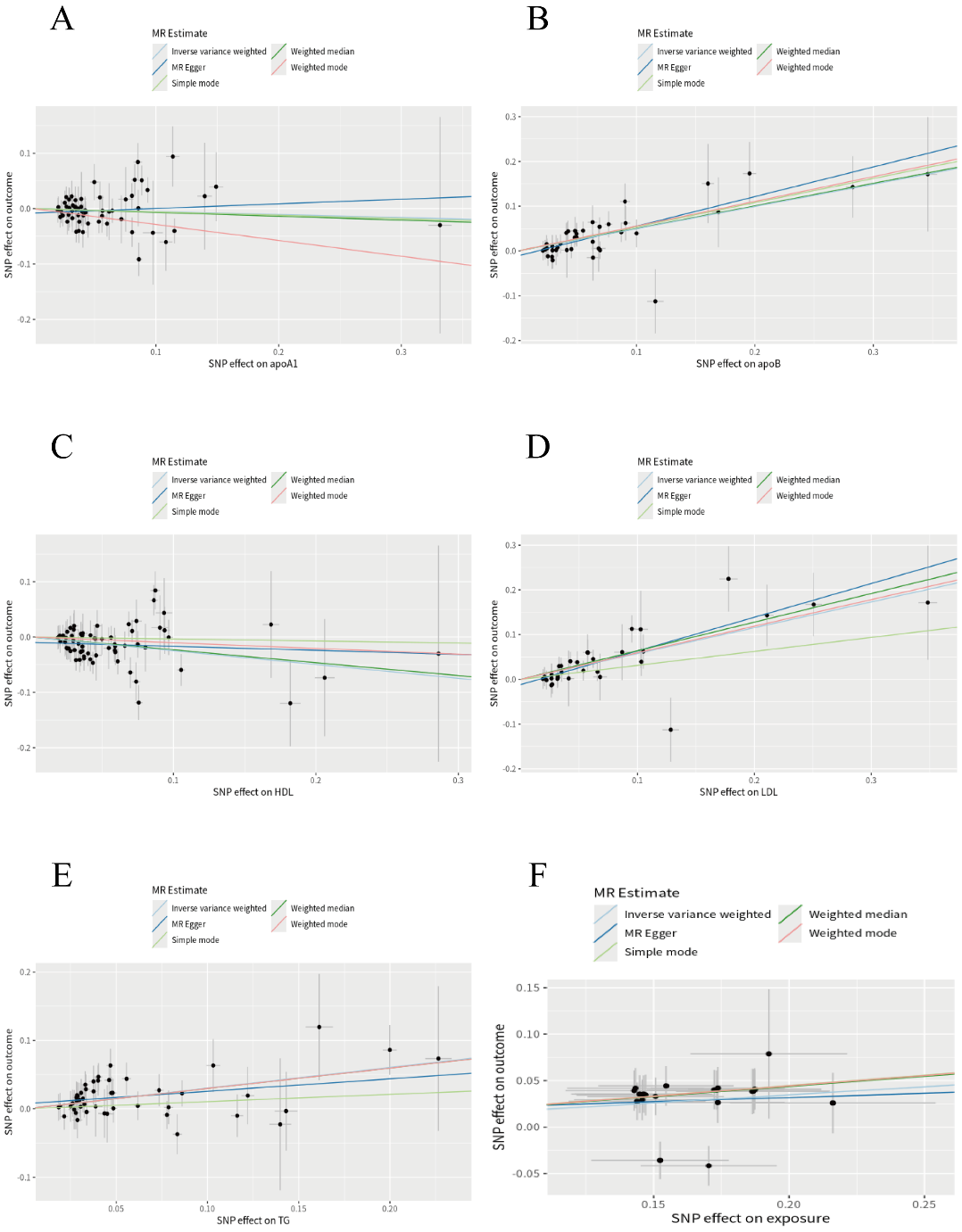


**Supplementary Figure 1.** Scatter plot using all IVs (discovery). (A) ApoA1 and CAC; (B) ApoB and CAC; (C) HDL-C and CAC; (D) LDL-C and CAC; (E) TG and CAC. (F) PCSK9-eqtl and CAC.


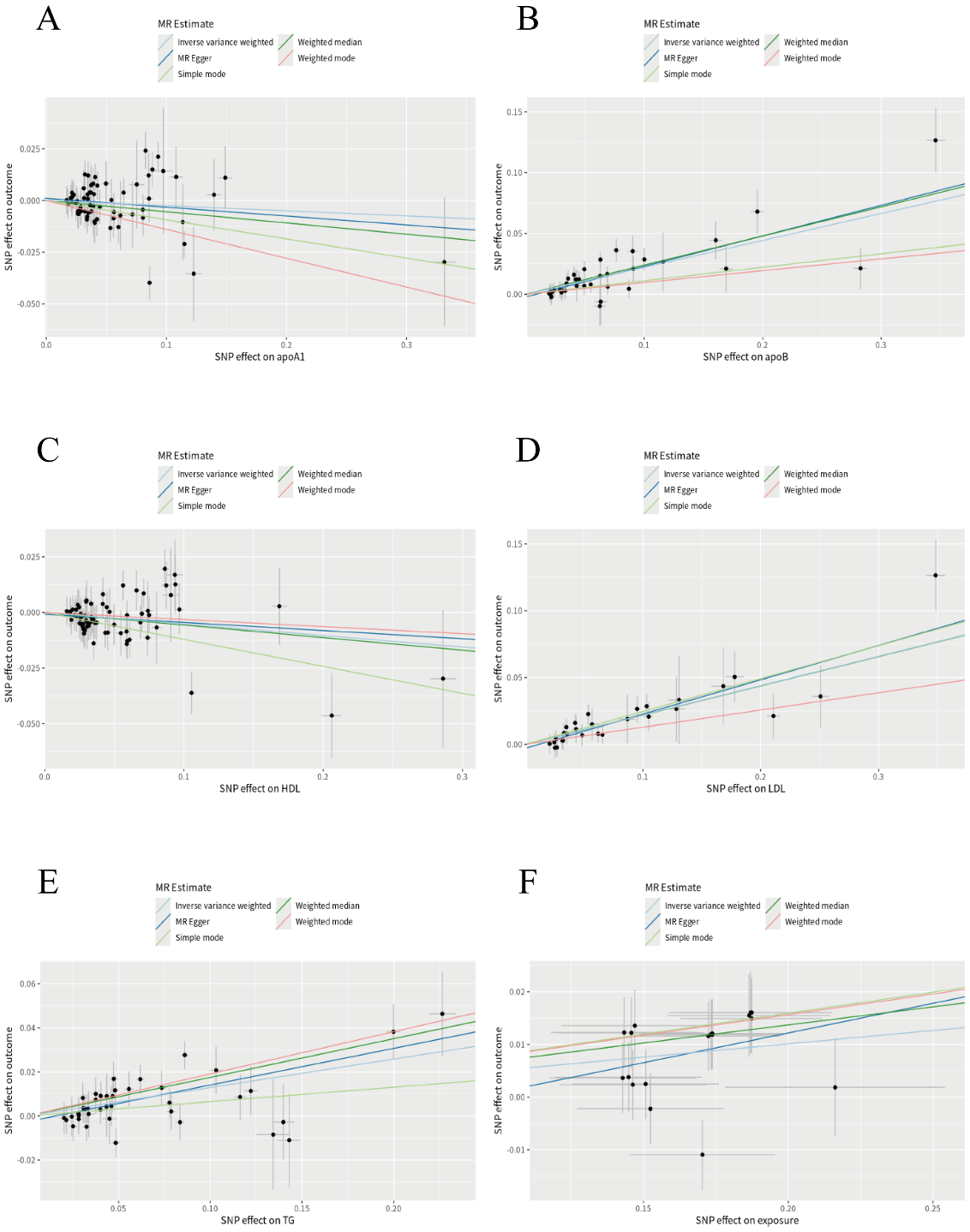


**Supplementary Figure 2.** Scatter plot using all IVs (replication). (A) ApoA1 and CAC; (B) ApoB and CAC; (C) HDL-C and CAC; (D) LDL-C and CAC; (E) TG and CAC. (F) PCSK9-eqtl and CAC.


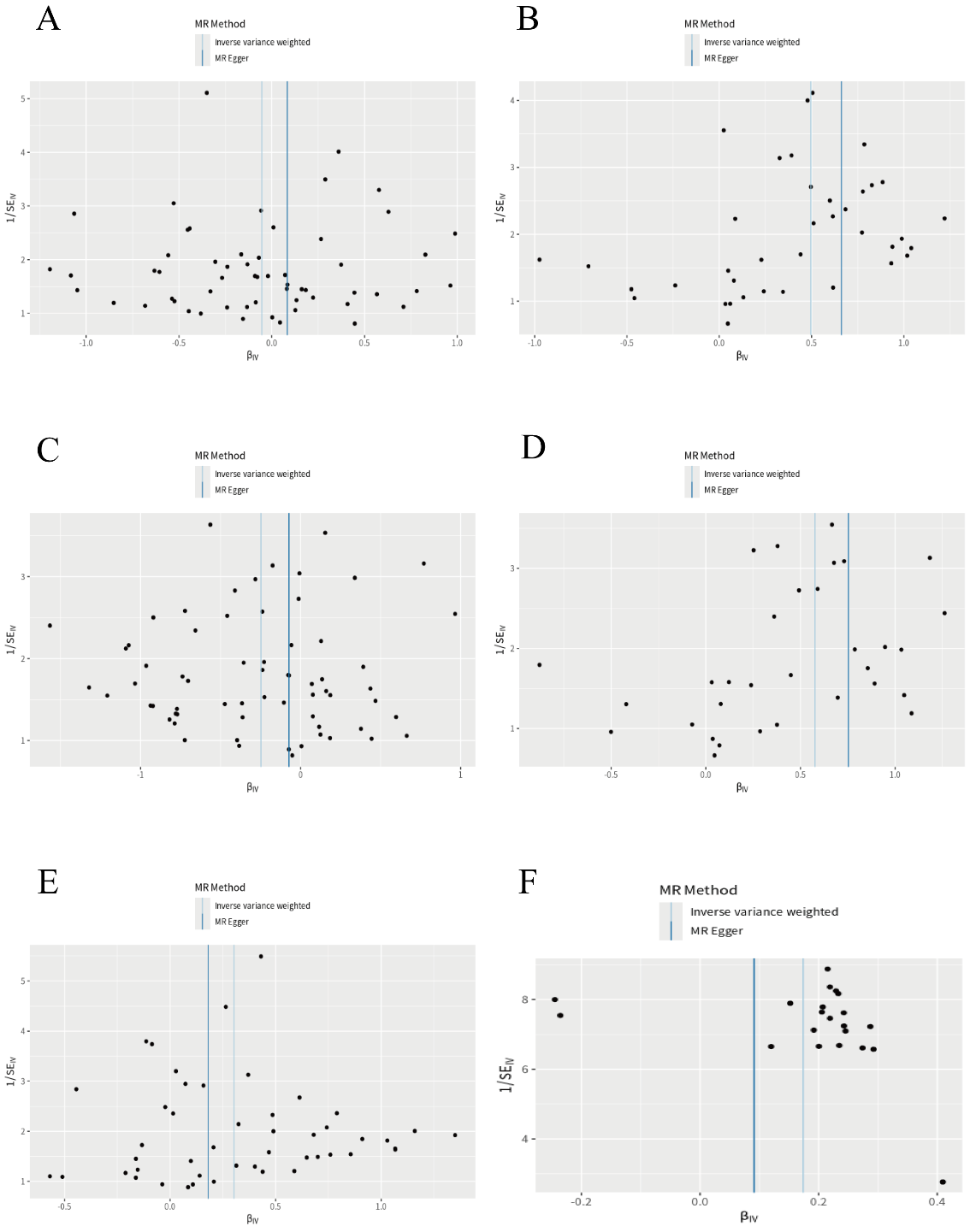


**Supplementary Figure 3.** Funnel plot for IVW and MR-Egger method(discovery). (A) ApoA1 and CAC; (B) ApoB and CAC; (C) HDL-C and CAC; (D) LDL-C and CAC; (E) TG and CAC. (F) PCSK9-eqtl and CAC.


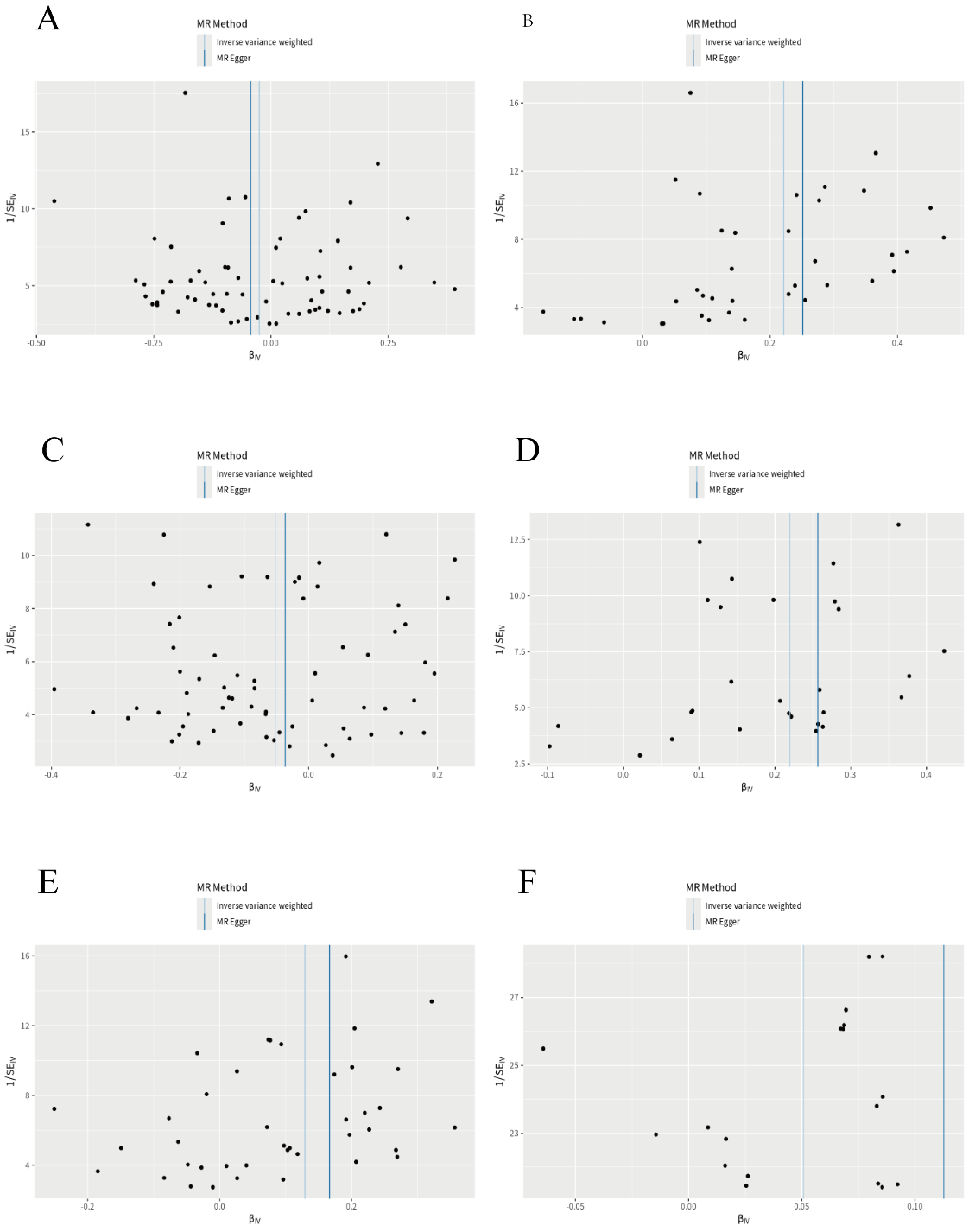


**Supplementary Figure 4.** Funnel plot for IVW and MR-Egger method(replication). (A) ApoA1 and CAC; (B) ApoB and CAC; (C) HDL-C and CAC; (D) LDL-C and CAC; (E) TG and CAC. (F) PCSK9-eqtl and CAC.


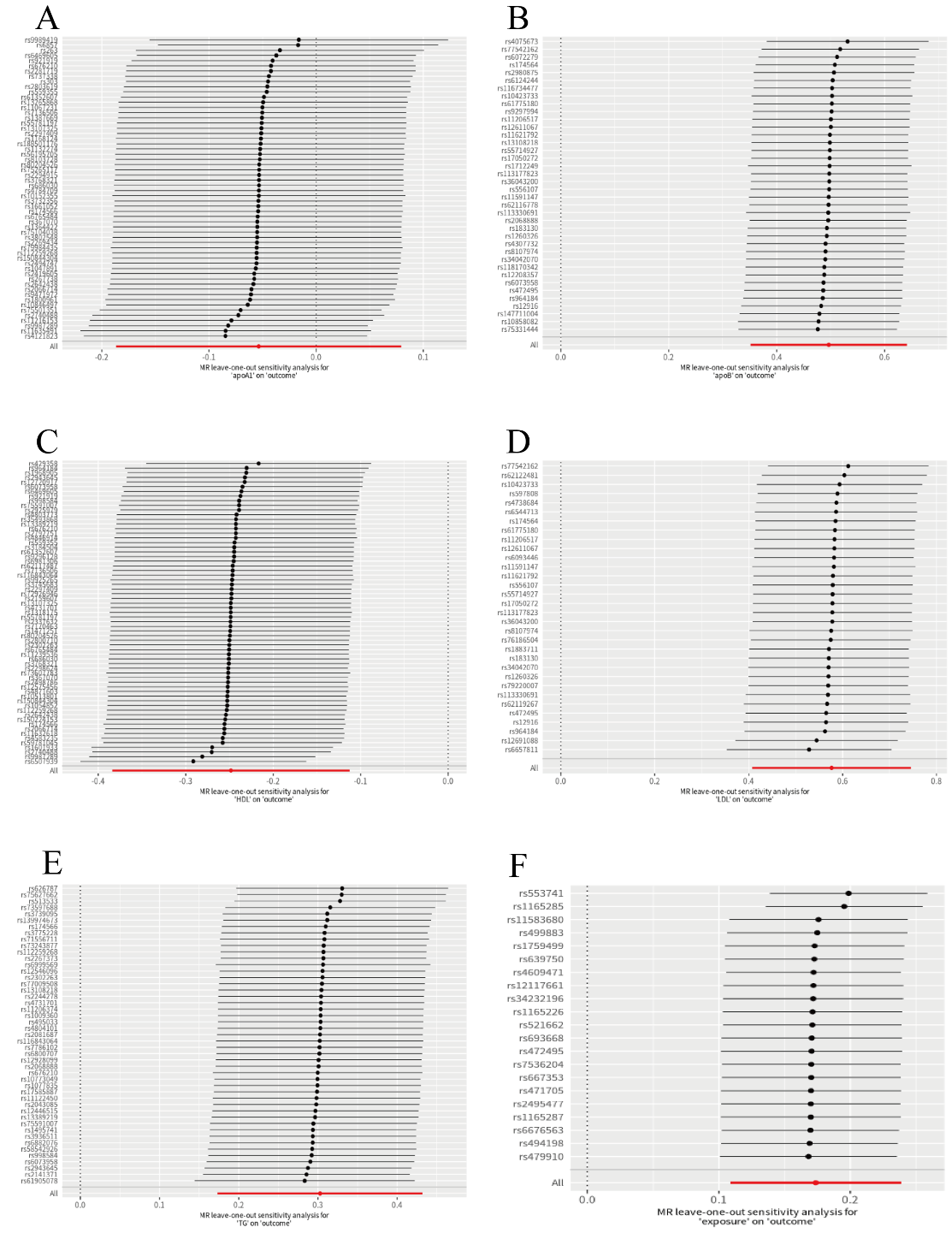


**Supplementary Figure 5.** Leave-one-out sensitivity analysis(discovery). (A) ApoA1 and CAC; (B) ApoB and CAC; (C) HDL-C and CAC; (D) LDL-C and CAC; (E) TG and CAC. (F) PCSK9-eqtl and CAC.


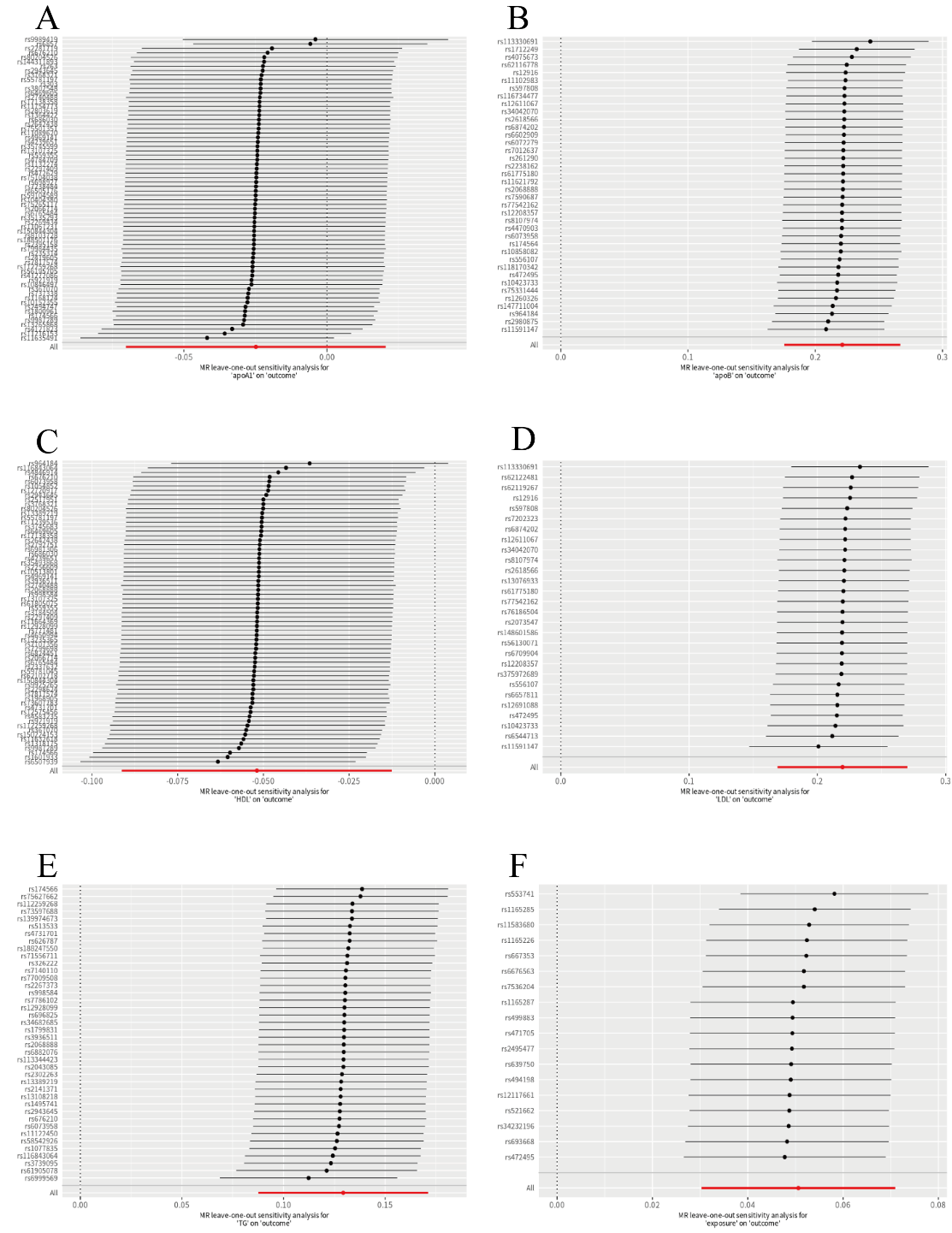


**Supplementary Figure 6.** Leave-one-out sensitivity analysis(replication). (A) ApoA1 and CAC; (B) ApoB and CAC; (C) HDL-C and CAC; (D) LDL-C and CAC; (E) TG and CAC. (F) PCSK9-eqtl and CAC.


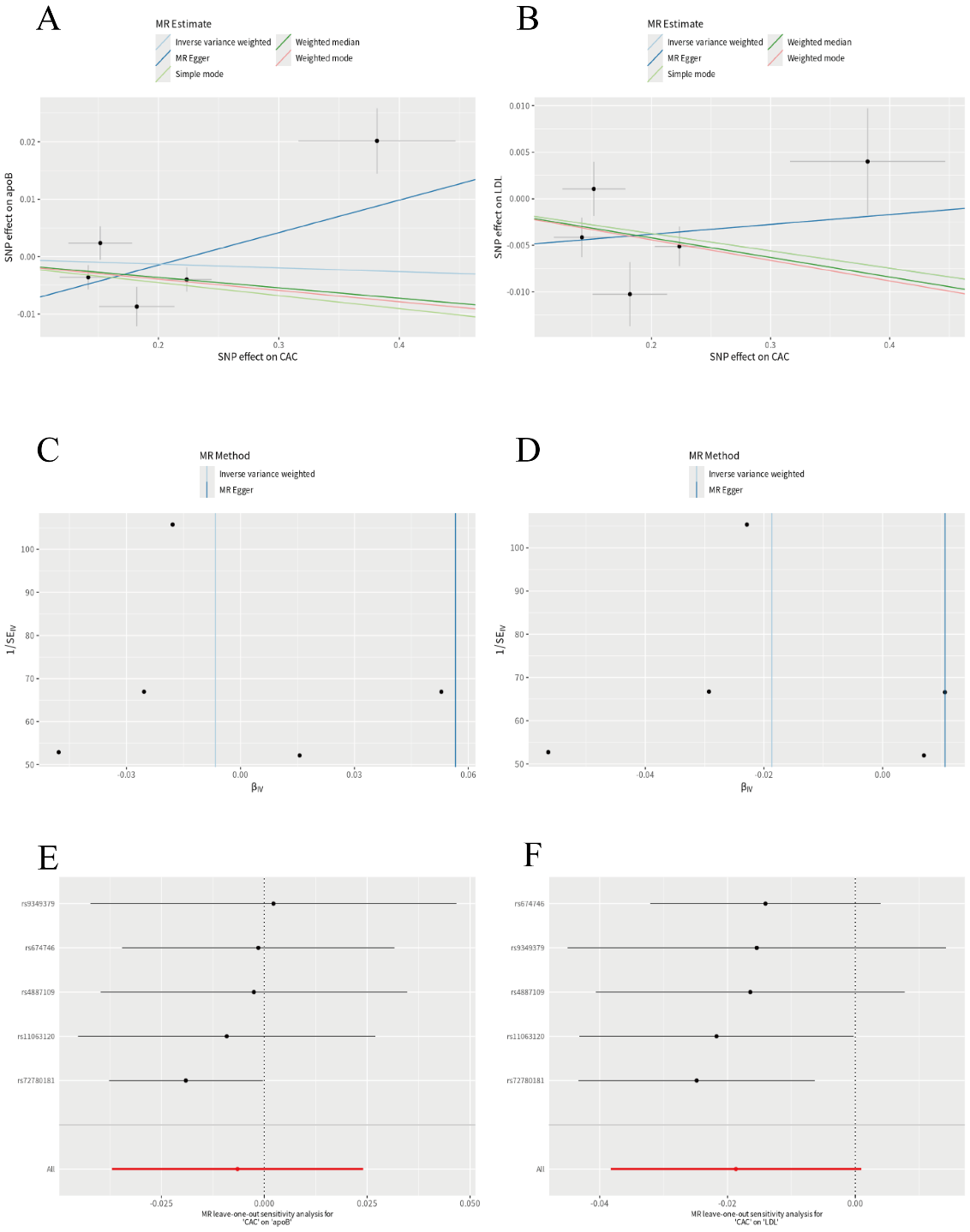


**Supplementary Figure 7** Reverse MR analysis(discovery). (A)CAC and ApoB(Scatter plot) (B) CAC and LDL-C(Scatter plot); (C) CAC and ApoB(Funnel plot) (D) CAC and LDL-C(Funnel plot); (E) CAC and ApoB(Leave-one-out). (F) CAC and LDL-C(Leave-one-out).


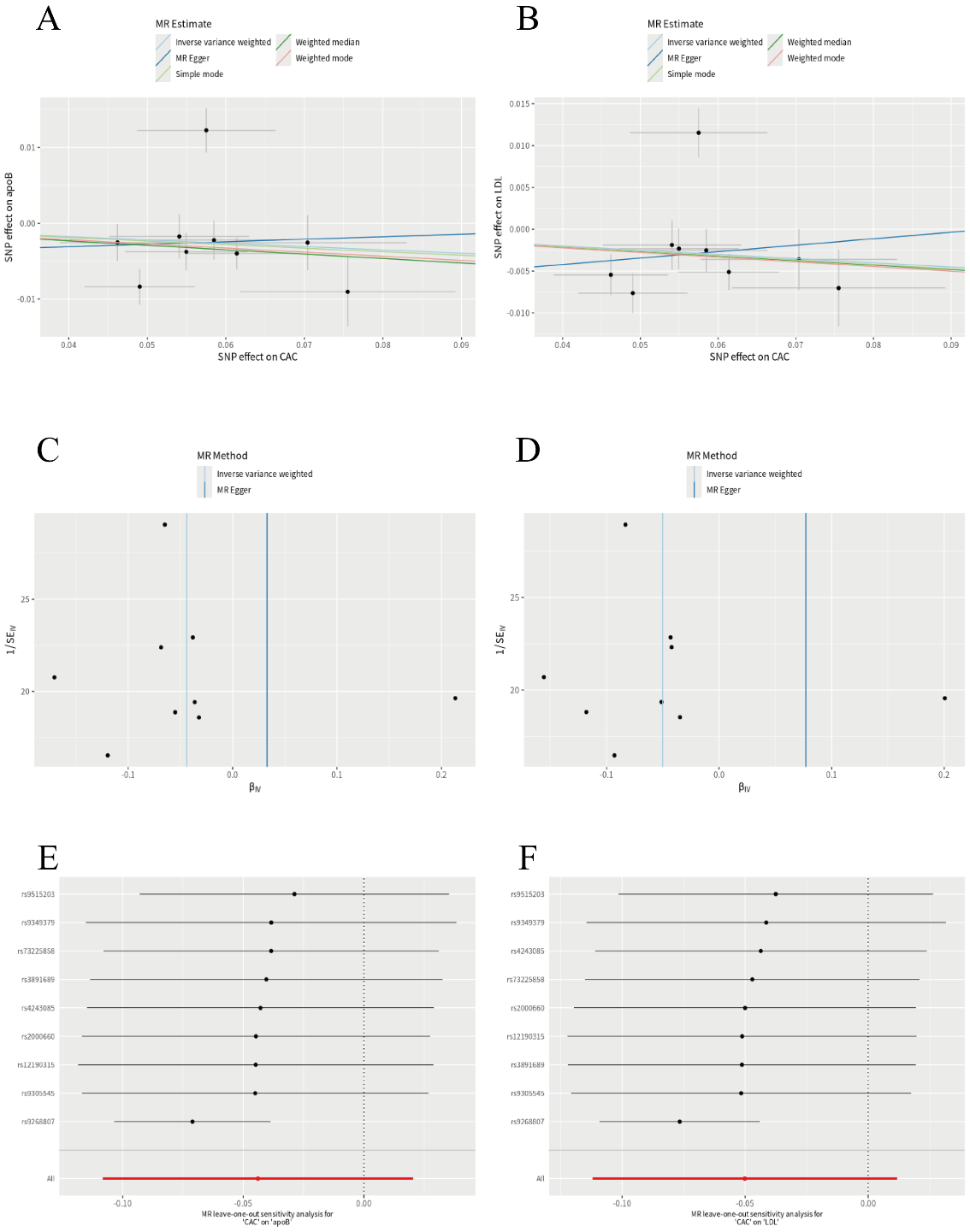


**Supplementary Figure 8** Reverse MR analysis(replication). (A)CAC and ApoB(Scatter plot) (B) CAC and LDL-C(Scatter plot); (C) CAC and ApoB(Funnel plot) (D) CAC and LDL-C(Funnel plot); (E) CAC and ApoB(Leave-one-out). (F) CAC and LDL-C(Leave-one-out).
